# Supplementary material for: Association of COVID-19 vaccines ChAdOx1 and BNT162b2 with major venous, arterial, or thrombocytopenic events: A population-based cohort study of 46 million adults in England
Source: PLoS Med. 2022 Feb 22;19(2):e1003926. doi: 10.1371/journal.pmed.1003926 (PMC8863280; doi:10.1371/journal.pmed.1003926)
Supplement: S1 Protocol — (PDF) [file pmed.1003926.s001.pdf]

Version history

V0.1	25/3/21	Out for comments
V0.2	1/4/21	Spencer Keene comments
V0.3	5/4/21	Cathie Sudlow comments, updated relevant phenotypes, added tables
V0.4	7/4/21	Munir Pirmohamed comments, Cathie Sudlow comments, updated phenotypes, added pre and post COVID analysis, added skeleton tables post Angela Wood comments, added thrombophilia to phenotypes, altering of DVT codes after Spencer Keene suggestions
V0.5	8/4/21	Altered follow up time periods to 1,2,3,4,8,12,20,39 weeks, updated to 4-character ICD-10 when possible, new COVID phenotypes matched to prior long COVID application
V0.6	9/4/21	Updated to include pregnancy related venous thrombosis codes and separate into a category, outcomes now in better table in appendix, updated tables and analyses
V0.7	11/4/21	Updated to include SAH, retinal infarction in list of codes, removed time since registration from population definition and changing practice
V0.8	12/4/21	Added instructions for follow up time from Angela Wood
V0.9	15/4/21	Aziz Sheikh comments, dates of follow up for COVID and vaccine populations, sensitivity date, added drug recoding from Venexia Walker
V0.10	26/4/21	Updated statistical methods, clarified COVID terminology, updated DVT phenotype to I80$ and ischaemic stroke to I63$ excluding I63.6, with further comments from Jonathan Sterne, Spencer Keene, Angela Wood. Updated ordering outcomes to first recorded only in SUS and ONS death rather than any position.
V0.11	27/4/21	Changes to spelling and grammar, added time from second vaccine
V1.0	27/4/21	To GitHub
V1.1	28/4/21	Changes to ischaemic stroke phenotype, added lower limb fracture phenotype, added more detail to modelling strategy after reviewing power available for various analyses
V1.2	5/5/21	Further details of statistical analyses, including stratification and inverse probability weighting of Cox models, approaches to control of confounding, dealing with missing data and subgroup analyses.

Lay summary

Some people have had illnesses related to blood clotting after COVID-19 vaccination. The illness that is of most concern is a very rare condition of clotting of the veins in unusual places like the brain and the gut, which has been associated with low levels of platelets, which are fragments of cells in our blood that form clots and help stop bleeding.

We will study all adults alive in England at the beginning of the COVID-19 pandemic (January 2020) with data included in the NHS Digital Trusted Research Environment. We will find out which people had a disease with blood clots in the arteries, like stroke or heart attack, or in the veins, like deep vein thrombosis, pulmonary embolism or clots in the veins of the brain or gut. We will also find out which of these people were diagnosed with low levels of platelets at the time of their blood clot.

We will compare the risk of developing one of these conditions among people who have had a COVID-19 vaccine with the risk in those who have not had a vaccine. We will account for differences between vaccinated and unvaccinated people (such as age, sex, ethnic group and previous medical history) that might affect the risk of these conditions. We will assess the risks of clotting events of different types, for different vaccines, in people who have had one or two vaccine doses, and for people with different characteristics. We will also compare the risk of these conditions following COVID-19 disease with the risk in people who do not have COVID-19.

This research is needed to provide reliable information about any risks associated with COVID-19 vaccines to medicine regulators, the UK Departments of Health, health professionals, and the public. Because clotting events are rare, it will be important to understand not only whether there is an increased risk with a COVID vaccine but also the size of any increased risk and whether it only applies to particular groups of people. It will also be important to understand how any risk compares with the benefits of the vaccine, which are known to be substantial.

AUTHORS

BHF Data Science Centre

TITLE

COVID-19 vaccination and disease and the risks of major venous and arterial vascular events

BACKGROUND

There have been reports of intracranial venous sinus thrombosis with intracranial haemorrhage, mesenteric thrombosis, thrombocytopenia and disseminated intravascular coagulation after Oxford-Astra Zeneca severe acute respiratory syndrome coronavirus 2 vaccination (henceforth 'COVID-19 vaccination').[1–3] Whether these events are caused by the vaccine is uncertain. It has been proposed that antibodies to platelet factor 4 lead to a haematological syndrome, analogous to heparin induced thrombocytopenia. Coronavirus-19 associated disease (henceforth 'COVID-19 disease') itself is associated with increased antibody-mediated procoagulant platelets.[4]

Vaccination in adulthood against other infections leads to a transient increase in inflammatory markers as the immune system begins an acute inflammatory response. Although higher levels of markers associated with inflammation are associated with a higher risk of myocardial infarction and stroke, there is little evidence that vaccination in adults increases the risk of myocardial infarction or stroke. A study based on UK electronic health records found no increase in incident myocardial infarction (MI) or stroke after influenza, tetanus, or pneumococcal vaccination;[5] this finding was replicated in a cohort of older American men who received pneumococcal vaccination.[6]

People who have been vaccinated tend to be in better health than those not vaccinated, at least at the time of vaccination. This may be why some case-control studies have found that pneumococcal vaccination is associated with a reduced risk of MI.[7] COVID vaccination was prioritised for older individuals and those with co-morbidities who may be in worse health (hence had a higher risk of thrombotic events), but those who were very unwell at the time of their vaccine appointment were encouraged to delay it.

Any increased risk of serious complications arising from COVID-19 vaccination needs to be balanced against the risk of these complications with COVID-19 disease, in the context of the rate of these events in individuals without COVID-19 vaccination or disease. Therefore, a comprehensive assessment of haemorrhagic and thrombotic vascular events after COVID-19 vaccination and COVID-19 disease, accounting as far as possible for confounding factors, is needed.

RESEARCH HYPOTHESES

1. There is a higher risk of venous or arterial thrombosis, or haemorrhage (particularly intracranial) after COVID-19 vaccination than before or without vaccination, that varies with vaccine type, and is associated with thrombocytopenia.
2. There is a higher risk of venous and arterial thrombosis or embolism after COVID-19 disease than before or without infection.

DATA SOURCES

NHS Digital TRE for England (up to latest release)

- Primary care data (GP Data for Pandemic Planning and Research via General Practice Extraction Service, GPES)
- Secondary Use Service (SUS) hospital data
- Pillar 1 and Pillar 2 COVID-19 infection laboratory testing data
- Hospital episode statistics Admitted Patient Care (HES APC)
- Office of National Statistics (ONS) death registration records
- Community dispensing data
- COVID-19 vaccination data

RESEARCH QUESTIONS

1. What is the age- and sex- specific incidence of each outcome event in 2018 and 2019 (before the pandemic) and in 2020 (during the pandemic)?
2. In people who have had COVID-19 vaccination compared with people who have not, are there higher rates (expressed as hazard ratios with time since vaccination) of fatal or non-fatal ischaemic stroke or MI (‘arterial); intracranial venous thrombosis, mesenteric or portal vein thrombosis, pulmonary embolism (PE) or deep vein thrombosis (DVT) (‘venous thromboembolism’); before and after adjustment for potential confounders (age, sex, ethnicity, socio-economic status and comorbidities)?
3. In people who have had COVID-19 disease compared with people who have not, are there higher rates (expressed as hazard ratios with time since COVID-19-disease) of fatal or non-fatal ischaemic stroke or MI (‘arterial); cerebral sinus thrombosis, mesenteric or portal vein thrombosis, PE or DVT (‘venous thromboembolism’); before and after adjustment for potential confounders?

STUDY POPULATION

Population for incidence of events during 2018-2020

Follow-up period: events in 2018, 2019 and 2020.

Whole population of England, estimated by mid-2019 population from ONS

Population for COVID analyses

Follow-up period: 1/1/20 to 7/12/20

Patients will be included if they meet ALL the following criteria:

- An age of ≥18 can be calculated on 1st January 2020;
- Known sex;
- Have a record in the primary care extract;
- Alive on 1st January 2020.

Exclude: a COVID-19 infection recorded prior to 1st January 2020.

Population for vaccine analyses

Follow up period: 8/12/20 until date of latest data release

Patients will be included if they meet ALL the following criteria:

- An age of ≥18 can be calculated on 8th December 2020;
- Known sex;
- Have a record in the primary care extract;
- Alive on 8th December 2020.

Exclude: a COVID-19 infection recorded prior to 1st January 2020.

EXPOSURES

PRIMARY – COVID-19 VACCINE

- Astra Zeneca
  - Batch number, dose number (first or second)
- Pfizer
  - Batch number, dose number (first or second)
- Similarly, for other vaccines when they become available

PRIMARY – COVID-19 DISEASE

- A positive PCR test and date of test available in laboratory data

SECONDARY – COVID-19 DISEASE

- **All detected:** positive test and/or any clinical code (i.e. 1,2, or 3 in table below)

		Test	
		+	-
**Any clinical code (COVID-19)**	+	1. Confirmed diagnosis	2. Clinical diagnosis only
	-	3. Asymptomatic [or asymptomatic code]	4. No known positive test or diagnosis

STATISTICAL METHODS

Follow up for each person will begin at the start of the follow up period and be censored at the first of: death; the outcome event, or the end of the follow up period. In analyses of the effects of individual vaccine types, follow up will be censored at receipt of another vaccine type. In analyses of first vaccine dose, follow up will be censored on the date of receipt of second dose. In analyses of second vaccine dose, follow up will start on the date of receipt of first dose.

We will split follow up time for each person into periods before and after exposure (COVID-19 vaccine or COVID-19 disease), and into time periods since exposure (Appendix 2). Outcome events of interest are listed below. We will tabulate numbers of outcome events, person-years of follow-up and rates of events before and with time since exposure.

We will fit survival models (Cox models or parametric survival models) in which time zero is defined as the calendar date of the start of follow up. This will ensure that all analyses account for changes with calendar time in rates of the outcome event. Using this approach, we will estimate hazard ratios for events of different types before and after exposure, and by time since exposure. For computational efficiency, Cox models will be fitted to datasets including all people with the outcome event and a random subset (for example, 10%) of people without the outcome event, with analyses incorporating inverse probability weights (for example, 10) for data from people without the outcome event, and confidence intervals derived using robust standard errors. All models will be stratified on region (so that risk sets are constructed within region, hence accounting for between-region variation in the baseline hazard). In sensitivity analyses we will confirm that results do not change markedly when we stratify on LSOA.

Potential confounding factors (factors that predict both exposure and outcome, listed below) will be based on data recorded on or before the start of follow up in each analysis. We will estimate (i) crude; (ii) age and sex adjusted and (iii) fully adjusted HRs (when there are sufficiently many outcome events). The following risk factors for venous thrombotic events will be included as confounders: sex, age, ethnicity, deprivation, anticoagulant prescription, combined oral contraceptive pill prescription, hormone replacement therapy prescription, history of pulmonary embolism or deep vein thrombosis, and history of coronavirus infection; and the following confounders for arterial thromboembolic events: sex, age, ethnicity,

deprivation, diabetes, hypertension, smoking, anti-platelet prescription, blood pressure lowering prescription, lipid lowering prescription, anticoagulant prescription, history of stroke, and history of MI. Other variables to be adjusted for will be selected from the list of potential confounders using a backwards stepwise procedure with p value threshold 0.2, from models using MI as an outcome (separately for males and females and for older and younger individuals). Variables selected in any model will be adjusted for in all models. The same selected variables will be adjusted for in models for all outcomes.

Missing data

All analyses will be "complete-case" analyses (note that many potential confounders are defined using the presence versus absence of specific codes in the EHRs, so by definition have no missing values). We will not use multiple imputation or indicators for missing data.

Subgroup analyses

We will estimate post-exposure hazard ratios:

- Following any vaccination, and separately after Pfizer, Oxford/Astra Zeneca and other vaccinations;
- Separately within age groups, for all people and in males and females;
- When there are sufficiently many outcome events, by separate calendar periods (e.g. before or after June 2020 between the two waves of COVID-19; month since 8th December 2020 (from the start of vaccination); before or after 18th March 2021 (the date on which a possible risk of thrombotic events associated with COVID-19 vaccination was first reported by the EMA).

When there are at least 400 outcome events, we will estimate post-exposure hazard ratios:

- Separately in people with and without a history of cardiovascular disease.
- Separately within subgroups of particular interest (see "effect modifiers") below.

These subgroup analyses will allow for different baseline hazard functions and differential confounding effects within subgroups, because associations between exposures and confounders and between confounders and outcomes may vary between subgroups.

When there are fewer than 400 outcome events, we will conduct sensitivity analyses in which the baseline hazard is additionally stratified by sex, and hazard ratios for males and females are estimated using the data on both sexes but allowing for exposure-sex interactions. Such an approach assumes the effect of covariates on the outcome to be the same in males and females, but avoids fitting a model including a large number of potential confounding variables to datasets with a small number of outcome events.

OUTCOMES (see appendix for code lists)

Each outcome is defined as the first event of that type following exposure in one of the following datasets: SUS, primary care or ONS death registry. For the primary analyses, we will use events in the primary position where recorded in SUS or death records, and in sensitivity analyses of the main results, events in any position. Some outcomes (largely venous) do not appear in the primary care data because the relevant codes are not included in the primary care extract available in the NHS Digital Trusted Research Environment, so will only be ascertained in SUS or death records.

Events will be defined as fatal if they are followed by death of any cause within 28 days, or are only recorded as fatal (i.e. reported only in death records). We will examine all events and fatal events in separate analyses. Lower limb fractures are included as an outcome that is unlikely to be affected by infection or vaccination.

Composite events:

- **Arterial events:** first of ischaemic stroke or stroke of unknown type or retinal infarction or myocardial infarction or other arterial thrombosis
- *Arterial events + thrombocytopenia* (i.e. any arterial event + any thrombocytopenia code present during in same spell), and no prior history of thrombocytopenia
- **Venous events:** first of pulmonary embolism or lower limb deep venous thrombosis or intracranial venous thrombosis or intracranial venous thrombosis during pregnancy or portal vein thrombus or other deep vein thrombosis
- *Venous events + thrombocytopenia* (i.e. both codes present during same spell) and no prior history of thrombocytopenia
- *ICVT + thrombocytopenia* (i.e. both codes present during same spell) and no prior history of thrombocytopenia

Individual events of different types

Arterial

- Incident myocardial infarction in primary care or SUS or ONS death
- Incident ischaemic stroke or stroke of unknown type or retinal infarction in primary care or SUS or ONS death
- Incident non-stroke non-MI arterial embolism in primary care or SUS or ONS death

Venous

- Pulmonary embolism in SUS or ONS death
- Deep vein thrombosis in SUS or ONS death
- Portal vein thrombosis in SUS or ONS death
- Other deep vein thrombosis in SUS or ONS death
- Thrombosis during pregnancy (excluding intracranial venous thrombosis) in SUS or ONS death
- Intracranial venous thrombosis during pregnancy in SUS or ONS death
- Intracranial venous thrombosis[8] in SUS or ONS death

Haematological

- Disseminated intravascular coagulation in SUS or ONS death
- Thrombotic thrombocytopenic purpura in SUS or ONS death
- Thrombocytopenia in SUS or ONS death

Other

- Intracerebral haemorrhage in primary care or SUS or ONS death
- Mesenteric thrombus in SUS or ONS death
- Spinal stroke in SUS or ONS death
- Lower limb fracture in SUS or ONS death

Date of onset defined as: of date of start of SUS or APC spell with event; OR date of GP consultation with event; OR death with event (whichever comes first)

POTENTIAL CONFOUNDERS

Defined on the inception date (defined henceforth as 1st January 2020 for COVID-19 disease analysis and 8th December 2020 for COVID-19 vaccine analysis), with a look back in GP and HES APC for each person:

- **Sex:** categorical, GP;
- **Age in years** continuous, at inception; GP;
- **Ethnicity** 5 categorical, most recent recorded prior to inception; GP and if missing from GP data then from HES;
- **Deprivation** continuous, most recent recorded prior to inception; HES or GP;
- **Region**: East of England, London, Midlands, NE and Yorkshire, North West, South East, South West, Scotland, Wales, most recent residence prior to inception; GP;
- **Consultation rate**: number of primary care contacts in the year prior to inception; GP;
- **Medications**: total number of medications by BNF chapters prescribed within three months prior to the inception date; GP;
- **Diabetes**: yes/not recorded from start of record to inception; GP or HES APC;
- **Depression**: yes/not recorded from start of record to inception; GP or HES APC;
- **Obesity**: yes/not recorded from start of record to inception or BMI>30; GP
- **Cancer** yes/not recorded from start of record to inception; GP or HES APC;
- **COPD** yes/not recorded from start of record to inception; GP or HES APC:
- **CKD** yes/not recorded from start of record to inception; GP or HES APC;
- **Liver disease**: yes/not recorded from start of record to inception; GP or HES APC;
- **Major Surgery** yes/not recorded from start of record to inception; GP or HES APC;
- **Hypertension** yes/not recorded from start of record to inception; GP or HES APC;
- **Dementia** yes/not recorded from start of record to date of linkage; GP or HES APC;
- **Smoking** current/ex-/never/unknown most recent prior to inception; GP;
- **Antiplatelet** yes/no/unknown (unknown if patient's GP practice not in prescription extract) BNF codes starting '0209' with at least one prescription within three months prior to the inception date; dispensing;
- **BP lowering** yes/no/unknown (unknown if patient's GP practice not in prescription extract) BNF codes starting with any of the following patterns provided below with at least one prescription within three months prior to the inception date, Aliskiren: '0205053A0' Alpha blockers: '020504' Beta blockers: '0204' (exclude: '0204000R0','0204000Q0') Calcium channel blockers: '020602' Centrally acting antihypertensives: '020502' (exclude: '0205020G','0205052AE') Potassium sparing diuretics: '020203' Thiazide diuretics: '020201' Vasodilator antihypertensives: '020501' Angiotensin-converting enzyme inhibitors: '0205051' Angiotensin-II receptor antagonists: '0205052'; dispensing;
- **Lipid lowering** yes/no/unknown (unknown if patient's GP practice not in prescription extract) BNF codes starting '0212' at least one prescription within three months prior to the inception date; dispensing;
- **Anticoagulant** yes/no/unknown (unknown if patient's GP practice not in prescription extract) BNF codes starting '020802' (exclude: '0208020I','0208020W') with at least one prescription within three months prior to the inception date; dispensing;
- **COCP** yes/no/unknown (unknown if patient's GP practice not in prescription extract) BNF codes starting '070301' with at least one prescription within three months prior to the inception date; dispensing;
- **HRT** yes/no/unknown (unknown if patient's GP practice not in prescription extract) BNF codes starting '0604011' with at least one prescription within three months prior to the inception date; dispensing;
- **History of stroke** yes/not recorded any stroke type (from 'outcomes above, ischaemic stroke, intracranial haemorrhage, or stroke of unknown type) recorded from start of record to inception; GP or HES APC;
- **History of MI**: yes/not recorded any MI type (from 'outcomes above) recorded from start of record to inception; GP or HES APC;

- **History of VT**: yes/not recorded and venous event (from 'outcomes above) recorded from start of record to inception; GP or HES APC;
- **History of thrombophilia**: yes/not recorded from start of record to inception, defined as ICD-10 D68.5 D68.6; acquired or inherited; GP or HES APC;
- **History of thrombocytopenia** (from outcomes above) yes/not recorded from start of record to inception; GP or HES APC;
- **History of coronavirus infection before vaccination**, defined as a positive PCR test or a clinical episode of COVID from 1st January 2020 until 8th December 2020 (only for vaccine analysis); GP or HES APC;

EFFECT MODIFIERS (see definitions in list of confounders above)

- Age — within age group categories
- Sex: — categorical;
- Ethnicity: — categorical;
- Medication: — yes/no for each of antiplatelet, BP lowering, lipid lowering, anticoagulant, COCP/HRT;
- Diabetes — yes/not known;
- Deprivation — fifths of deprivation distribution;
- Calendar month — and before and after 18th March
- Prior CVD events — yes/not known;
- Prior low platelets — yes/not known;

REFERENCES

1. Cines DB, Bussel JB. SARS-CoV-2 Vaccine-Induced Immune Thrombotic Thrombocytopenia. *N Engl J Med* [Internet]. 2021 [cited 2021 Apr 26];NEJMe2106315. Available from: http://www.ncbi.nlm.nih.gov/pubmed/33861524

2. Schultz NH, Sørvoll IH, Michelsen AE, Munthe LA, Lund-Johansen F, Ahlen MT, Wiedmann M, Aamodt A-H, Skattør TH, Tjønnfjord GE, Holme PA. Thrombosis and Thrombocytopenia after ChAdOx1 nCoV-19 Vaccination. *N Engl J Med* [Internet]. 2021 [cited 2021 Apr 26];NEJMoa2104882. Available from: http://www.nejm.org/doi/10.1056/NEJMoa2104882

3. Greinacher A, Thiele T, Warkentin TE, Weisser K, Kyrle PA, Eichinger S. Thrombotic Thrombocytopenia after ChAdOx1 nCov-19 Vaccination. *N Engl J Med* [Internet]. 2021 [cited 2021 Apr 26];NEJMoa2104840. Available from: http://www.nejm.org/doi/10.1056/NEJMoa2104840

4. Althaus K, Marini I, Zlamal J, Pelzl L, Singh A, Häberle H, Mehrländer M, Hammer S, Schulze H, Bitzer M, Malek N, Rath D, Bösmüller H, Nieswandt B, Gawaz M, Bakchoul T, Rosenberger P. Antibody-induced procoagulant platelets in severe COVID-19 infection. *Blood* [Internet]. 2021 [cited 2021 Apr 8];137:1061–1071. Available from: http://ashpublications.org/blood/article-pdf/137/8/1061/1800923/bloodbld2020008762.pdf

5. Smeeth L, Thomas SL, Hall AJ, Hubbard R, Farrington P, Vallance P. Risk of Myocardial Infarction and Stroke after Acute Infection or Vaccination. *N Engl J Med* [Internet]. 2004 [cited 2021 Mar 25];351:2611–2618. Available from: https://pubmed.ncbi.nlm.nih.gov/15602021/

6. Tseng HF, Slezak JM, Quinn VP, Sy LS, Van Den Eeden SK, Jacobsen SJ. Pneumococcal vaccination and risk of acute myocardial infarction and stroke in men. *JAMA - J Am Med Assoc* [Internet]. 2010 [cited 2021 Mar 25];303:1699–1706. Available from: https://jamanetwork.com/

7. Lamontagne F, Garant MP, Carvalho JC, Lanthier L, Smieja M, Pilon D. Pneumococcal vaccination and risk of myocardial infarction. *CMAJ* [Internet]. 2008 [cited 2021 Mar 25];179:773–777. Available from: /pmc/articles/PMC2553874/

8. Handley JD, Emsley HCA. Validation of ICD-10 codes shows intracranial venous thrombosis incidence to be higher than previously reported. *Heal Inf Manag J* [Internet]. 2020 [cited 2021 Mar 25];49:58–61. Available from: https://pubmed.ncbi.nlm.nih.gov/30563370/

Appendix 1 : outcome definitions

Composite events, composed of more than one phenotype	
Arterial event	First of *ischaemic stroke* or *stroke of unknown type* or *myocardial infarction or retinal infarction* or *other arterial embolism*
Arterial events + thrombocytopenia	Any *arterial event* + any *thrombocytopenia* code present during in same spell and no prior history of *thrombocytopenia*
Venous event	first of *pulmonary embolism* or *deep venous thrombosis* or *other deep vein thrombosis* or *portal vein thrombosis* or *intracranial venous thrombosis*
Venous events + thrombocytopenia	Any *venous event* + any *thrombocytopenia* code present during in same spell and no prior history of *thrombocytopenia*

Individual events	Phenotype	Code	description
Arterial	Incident myocardial infarction	I21*	Acute myocardial infarction
Arterial	Incident myocardial infarction	I22*	Subsequent myocardial infarction
Arterial	Incident myocardial infarction	I23*	Certain current complications following acute myocardial infarction
Arterial	Incident myocardial infarction	primary care codes, type=1	SNOMED codes
Arterial	Retinal infarction	H34*	Retinal vascular occlusion
Arterial	Ischaemic stroke	I63*	Cerebral infarction, excluding I63.6
Arterial	Ischaemic stroke	primary care codes type=1	SNOMED codes
Arterial	Stroke of unknown type	I64*	Stroke, not specified as haemorrhage or infarction
Arterial	Stroke of unknown type	Primary care codes =1	SNOMED codes
Arterial	Stroke, subarachnoid haemorrhage	I60*	Nontraumatic subarachnoid haemorrhage
Arterial	Other arterial embolism	I74*	Arterial embolism and thrombosis
Venous	Pulmonary embolism	I26.0	Pulmonary embolism without mention of acute cor pulmonale
Venous	Pulmonary embolism	I26.9	Pulmonary embolism with mention of acute cor pulmonale
Venous	Deep vein thrombosis	I80*	Phlebitis and thrombophlebitis of other sites
Venous	Portal vein thrombosis	I81*	Portal vein thrombosis
Venous	Other deep vein thrombosis	I82.0	Budd Chiari Syndrome
Venous	Other deep vein thrombosis	I82.2	Embolism and thrombosis of vena cava
Venous	Other deep vein thrombosis	I82.3	Embolism and thrombosis of renal vein
Venous	Other deep vein thrombosis	I82.8	Embolism and thrombosis of other specified veins
Venous	Other deep vein thrombosis	I82.9	Embolism and thrombosis of unspecified vein
Venous	Thrombosis during pregnancy and puerperium	O22.3	Deep phlebothrombosis in pregnancy
Venous	Thrombosis during pregnancy and puerperium	O87.1	Deep phlebothrombosis in the puerperium

Venous	Thrombosis during pregnancy and puerperium	O87.9	Venous complication in the puerperium, unspecified
Venous	Thrombosis during pregnancy and puerperium	O88.2	Obstetric blood-clot embolism
Venous	Cerebral venous thrombosis during pregnancy and puerperium	O22.5	Cerebral venous thrombosis in pregnancy
Venous	Cerebral venous thrombosis during pregnancy and puerperium	O87.3	Cerebral venous thrombosis in the puerperium
Venous	Cerebral venous thrombosis	G08*	Intracranial and intraspinal phlebitis and thrombophlebitis
Venous	Cerebral venous thrombosis	I67.6	Nonpyogenic thrombosis of intracranial venous system
Venous	Cerebral venous thrombosis	I63.6	Cerebral infarction due to cerebral venous thrombosis, nonpyogenic
Haematological	Thrombocytopenia	D69.3	Idiopathic thrombocytopenic purpura
Haematological	Thrombocytopenia	D69.4	Other primary thrombocytopenia
Haematological	Thrombocytopenia	D69.5	Secondary thrombocytopenia
Haematological	Thrombocytopenia	D69.6	Thrombocytopenia, unspecified
Haematological	Disseminated intravascular coagulation	D65*	Disseminated intravascular coagulation
Haematological	Thrombotic thrombocytopenic purpura	M31.1	Thrombotic microangiopathy
Uncertain classification	Mesenteric thrombus	K55.9	Vascular disorders of intestine, unspecified (arterial or venous)
Uncertain classification	Mesenteric thrombus	K55.0	Acute vascular disorders of intestine (arterial or venous)
Uncertain classification	Intracerebral haemorrhage	I61*	Intracerebral haemorrhage
Uncertain classification	Spinal stroke	G95.1	Vascular myelopathies (arterial or venous)
Fractures	Lower limb fracture	S720	Fracture of neck of femur
Fractures	Lower limb fracture	S721	Pertrochanteric fracture
Fractures	Lower limb fracture	S723	Fracture of shaft of femur
Fractures	Lower limb fracture	S724	Fracture of lower end of femur
Fractures	Lower limb fracture	S727	Multiple fractures of femur
Fractures	Lower limb fracture	S728	Fractures of other parts of femur
Fractures	Lower limb fracture	S729	Fracture of femur, part unspecified
Fractures	Lower limb fracture	S820	Fracture of patella
Fractures	Lower limb fracture	S821	Fracture of upper end of tibia (and fibula)
Fractures	Lower limb fracture	S822	Fracture of shaft of tibia (and fibula)
Fractures	Lower limb fracture	S823	Fracture of lower end of tibia (and fibula)
Fractures	Lower limb fracture	S824	Fracture of fibula alone
Fractures	Lower limb fracture	S825	Fracture of medial malleolus
Fractures	Lower limb fracture	S826	Fracture of lateral malleolus
Fractures	Lower limb fracture	S827	Multiple fractures of lower leg
Fractures	Lower limb fracture	S828	Fractures of other parts of lower leg
Fractures	Lower limb fracture	S829	Fracture of lower leg, part unspecified
Fractures	Lower limb fracture	S920	Fracture of calcaneus
Fractures	Lower limb fracture	S921	Fracture of talus
Fractures	Lower limb fracture	S922	Fracture of other tarsal bone(s)

Fractures	Lower limb fracture	S923	Fracture of metatarsal bone
Fractures	Lower limb fracture	S927	Multiple fractures of foot
Fractures	Lower limb fracture	S929	Fracture of foot, unspecified
Fractures	Lower limb fracture	T12	Lower limb fracture
Fractures	Lower limb fracture	T025	Fractures involving multiple regions of both lower limbs
Fractures	Lower limb fracture	T023	Fractures involving multiple regions of one lower limb

Appendix 2: splitting follow up according to time since the start of follow up and since exposure.

Definitions
Time scale – days since 1/1/2020 (for analyses examining hazard ratios after COVID-19 disease) or days since 8/12/2020 (for analyses examining hazard ratios after COVID-19 vaccination)

Outcome of interest – time to event D (T_D, I_D)

Exposure of interest – binary exposure E (COVID disease or COVID vaccination) measured at T_E with indicator I_E, parameterised as days since T_E, categorised for example into E1 = 0-13.999 E2=14-27.999; E3=28-41.999; E4=42-55.999; E5=56-70 days (although time interval may change depending on the number of events of different types)

Administrative Censoring time – set as day T_C

For <u>individuals without exposure and without event</u> then T_D=T_C, I_D = 0, T_E=T_C, I_E=0 (e.g., individual 1 in table below)

For <u>individuals without exposure and with event at time *t*</u> then T_D=*t*, I_D = 1, T_E=*t*, I_E=0 (e.g. individual 2 in table below)

For <u>individuals with exposure at T_E and without event</u> then: (1) split follow-up time at T_E, and (2) split follow-up time>T_E at T_E+14; T_E+28; T_E+42; T_E+56 and then censor at earliest of T_E+70 or T_C (e.g., individual 3 in table below)

For <u>individuals with exposure at T_E and event at T_D</u>, then first (1) split follow-up time at T_E, and then (2) split follow-up time>T_E at T_E+14; T_E+28; T_E+42; T_E+56 and then censor at earliest of T_E+70 or T_D (e.g., individual 4 in table below)

In example I have set T_C = 300

id	T_E	T_D	T_C	T0	T1	I_E	I_D	E1	E2	E3	E4	E5
1	300	300	300	0	300	0	0	0	0	0	0	0
2	47	47	300	0	47	0	1	0	0	0	0	0
3	35	300	300	0	35	0	0	0	0	0	0	0
3	35	300	300	35	49	1	0	1	0	0	0	0
3	35	300	300	49	63	1	0	0	1	0	0	0
3	35	300	300	63	77	1	0	0	0	1	0	0
3	35	300	300	77	91	1	0	0	0	0	1	0
3	35	300	300	91	105	1	0	0	0	0	0	1
4	105	136	300	0	105	0	0	0	0	0	0	0
4	105	136	300	105	129	1	0	1	0	0	0	0
4	105	136	300	129	136	1	1	0	1	0	0	0

Cox model in R:
Coxph(Surv(T0, T1, I_D) ~ E1+E2+E3+E4+E5)
