# Supplementary material for: Association of COVID-19 vaccines ChAdOx1 and BNT162b2 with major venous, arterial, or thrombocytopenic events: A population-based cohort study of 46 million adults in England
Source: PLoS Med. 2022 Feb 22;19(2):e1003926. doi: 10.1371/journal.pmed.1003926 (PMC8863280; doi:10.1371/journal.pmed.1003926)
Supplement: S1 Table — Estimates for (a) age-stratified risk of venous and arterial events during follow-up; (b) age-stratified incidence of events pre- and post-first ChAdOx1-S; (c) age-stratified incidence of events pre- and post-first BNT162b2; (d) age- and sex-stratified HR of events 1–28 and >28 days post-ChAdOx1-S vaccine; and (e) age- and sex-stratified HR of events 1–28 and >28 days post-ChAdOx1-S vaccine BNT162b2 vaccine. HR, hazard ratio. (PDF) [file pmed.1003926.s003.pdf]

**S1 Table (a). Numbers of patients analysed and (in parentheses) risk per 100,000 people for the whole population during follow up of venous and arterial events and thrombocytopenia (TCP), overall and according to age.**

| | | Overall | | | | Age, <70 | | | | Age, ≥70 | | | |
|---|---|---|---|---|---|---|---|---|---|---|---|---|---|
| | | All | Venous | Arterial | TCP | All | Venous | Arterial | TCP | All | Venous | Arterial | TCP |
| N | | 46,162,942 | 20,903 (45.3) | 87,251 (189) | 1,926 (4.2) | 38,744,171 | 11,835 (30.5) | 38,283 (98.8) | 1,167 (3.0) | 7,418,771 | 9,068 (122) | 48,968 (660) | 759 (10.2) |
| Sex | Male | 22,765,779 | 10,358 (45.5) | 51,851 (228) | 986 (4.3) | 19,425,454 | 6,406 (33.0) | 26,549 (137) | 555 (2.9) | 3,340,325 | 3,952 (118) | 25,302 (757) | 431 (12.9) |
| | Female | 23,397,163 | 10,545 (45.1) | 35,400 (151) | 940 (4.0) | 19,318,717 | 5,429 (28.1) | 11,734 (60.7) | 612 (3.2) | 4,078,446 | 5,116 (125) | 23,666 (580) | 328 (8.0) |
| Age | 18 - 29 | 8,821,973 | 781 (8.9) | 400 (4.5) | 143 (1.6) | 8,821,973 | 781 (8.9) | 400 (4.5) | 143 (1.6) | | | | |
| | 30 - 49 | 16,131,025 | 3,544 (22.0) | 6,424 (39.8) | 338 (2.1) | 16,131,025 | 3,544 (22.0) | 6,424 (39.8) | 338 (2.1) | | | | |
| | 50 - 69 | 13,791,173 | 7,510 (54.5) | 31,459 (228) | 686 (5.0) | 13,791,173 | 7,510 (54.5) | 31,459 (228) | 686 (5.0) | | | | |
| | 70 - 79 | 4,674,973 | 4,820 (103) | 22,249 (476) | 418 (8.9) | | | | | 4,674,973 | 4,820 (103) | 22,249 (476) | 418 (8.9) |
| | 80+ | 2,743,798 | 4,248 (155) | 26,719 (974) | 341 (12.4) | | | | | 2,743,798 | 4,248 (155) | 26,719 (974) | 341 (12.4) |
| Ethnicity | Asian or Asian British | 3,718,442 | 599 (16.1) | 5,325 (143) | 152 (4.1) | 3,467,223 | 475 (13.7) | 3,378 (97.4) | 125 (3.6) | 251,219 | 124 (49.4) | 1,947 (775) | 27 (10.7) |
| | Black or Black British | 1,634,470 | 654 (40.0) | 2,086 (128) | 58 (3.5) | 1,525,603 | 513 (33.6) | 1,384 (90.7) | 45 (2.9) | 108,867 | 141 (130) | 702 (645) | 13 (11.9) |
| | Mixed | 712,534 | 182 (25.5) | 642 (90.1) | 22 (3.1) | 682,037 | 153 (22.4) | 426 (62.5) | 19 (2.8) | 30,497 | 29 (95.1) | 216 (708) | <5 (9.8) |
| | Other ethnic groups | 1,497,133 | 217 (14.5) | 1,072 (71.6) | 39 (2.6) | 1,427,187 | 162 (11.4) | 678 (47.5) | 33 (2.3) | 69,946 | 55 (78.6) | 394 (563) | <10 (8.6) |
| | White | 36,446,855 | 18,995 (52.1) | 77,118 (212) | 1,643 (4.5) | 29,619,868 | 10,362 (35.0) | 31,821 (107) | 939 (3.2) | 6,826,987 | 8,633 (126) | 45,297 (663) | 704 (10.3) |
| | Unknown or missing | 2,153,508 | 256 (11.9) | 1,008 (46.8) | 12 (0.6) | 2,022,253 | 170 (8.4) | 596 (29.5) | 6 (0.3) | 131,255 | 86 (65.5) | 412 (314) | <10 (4.6) |
| Deprivation[1] | 1 - 2 | 9,118,746 | 4,565 (50.1) | 18,830 (206) | 365 (4.0) | 8,069,275 | 3,064 (38.0) | 10,493 (130) | 272 (3.4) | 1,049,471 | 1,501 (143) | 8,337 (794) | 93 (8.9) |
| | 3 - 4 | 9,567,633 | 4,105 (42.9) | 17,436 (182) | 370 (3.9) | 8,273,028 | 2,454 (29.7) | 8,370 (101) | 234 (2.8) | 1,294,605 | 1,651 (128) | 9,066 (700) | 136 (10.5) |
| | 5 - 6 | 9,355,549 | 4,091 (43.7) | 17,642 (189) | 392 (4.2) | 7,784,840 | 2,235 (28.7) | 7,263 (93.3) | 221 (2.8) | 1,570,709 | 1,856 (118) | 10,379 (661) | 171 (10.9) |
| | 7 - 8 | 9,076,313 | 4,146 (45.7) | 16,939 (187) | 381 (4.2) | 7,362,375 | 2,128 (28.9) | 6,334 (86.0) | 221 (3.0) | 1,713,938 | 2,018 (118) | 10,605 (619) | 160 (9.3) |
| | 9 - 10 | 8,772,902 | 3,926 (44.8) | 16,024 (183) | 406 (4.6) | 7,000,538 | 1,908 (27.3) | 5,602 (80.0) | 210 (3.0) | 1,772,364 | 2,018 (114) | 10,422 (588) | 196 (11.1) |
| Smoking status | Current | 7,814,045 | 3,079 (39.4) | 16,115 (206) | 260 (3.3) | 7,287,225 | 2,492 (34.2) | 11,811 (162) | 219 (3.0) | 526,820 | 587 (111) | 4,304 (817) | 41 (7.8) |
| | Former | 10,623,072 | 6,930 (65.2) | 31,768 (299) | 581 (5.5) | 7,826,062 | 3,233 (41.3) | 11,703 (150) | 277 (3.5) | 2,797,010 | 3,697 (132) | 20,065 (717) | 304 (10.9) |
| | Never | 25,722,494 | 10,667 (41.5) | 38,701 (150) | 1,053 (4.1) | 21,674,974 | 5,929 (27.4) | 14,346 (66.2) | 643 (3.0) | 4,047,520 | 4,738 (117) | 24,355 (602) | 410 (10.1) |
| Medical history | Stroke | 727,218 | 1,020 (140) | 21,489 (2,955) | 104 (14.3) | 286,650 | 323 (113) | 8,147 (2,842) | 40 (14.0) | 440,568 | 697 (158) | 13,342 (3,028) | 64 (14.5) |
| | Myocardial infarction | 1,189,182 | 1,436 (121) | 24,212 (2,036) | 158 (13.3) | 485,506 | 436 (89.8) | 10,486 (2,160) | 46 (9.5) | 703,676 | 1,000 (142) | 13,726 (1,951) | 112 (15.9) |
| | DVT or PE | 603,351 | 3,263 (541) | 4,050 (671) | 142 (23.5) | 341,788 | 1,919 (561) | 1,377 (403) | 76 (22.2) | 261,563 | 1,344 (514) | 2,673 (1,022) | 66 (25.2) |
| | Thrombophilia | 44,593 | 146 (327) | 202 (453) | 23 (51.6) | 38,136 | 126 (330) | 144 (378) | 21 (55.1) | 6,457 | 20 (310) | 58 (898) | <5 (31.0) |
| | Coronavirus infection[2] | 1,284,984 | 1,131 (88.0) | 4,167 (324) | 101 (7.9) | 1,124,135 | 576 (51.2) | 1,642 (146) | 63 (5.6) | 160,849 | 555 (345) | 2,525 (1,570) | 38 (23.6) |
| | Diabetes | 4,069,412 | 3,654 (89.8) | 25,595 (629) | 423 (10.4) | 2,460,649 | 1,509 (61.3) | 9,949 (404) | 205 (8.3) | 1,608,763 | 2,145 (133) | 15,646 (973) | 218 (13.6) |
| | Depression | 9,516,020 | 6,272 (65.9) | 24,146 (254) | 500 (5.3) | 8,040,590 | 4,077 (50.7) | 12,859 (160) | 348 (4.3) | 1,475,430 | 2,195 (149) | 11,287 (765) | 152 (10.3) |
| | Obesity | 10,918,274 | 8,863 (81.2) | 31,194 (286) | 679 (6.2) | 8,757,004 | 5,430 (62.0) | 15,848 (181) | 411 (4.7) | 2,161,270 | 3,433 (159) | 15,346 (710) | 268 (12.4) |
| | Cancer | 6,609,691 | 5,674 (85.8) | 17,827 (270) | 786 (11.9) | 4,854,822 | 2,583 (53.2) | 4,462 (91.9) | 412 (8.5) | 1,754,869 | 3,091 (176) | 13,365 (762) | 374 (21.3) |
| | COPD | 1,582,366 | 2,497 (158) | 12,323 (779) | 182 (11.5) | 714,880 | 877 (123) | 3,839 (537) | 76 (10.6) | 867,486 | 1,620 (187) | 8,484 (978) | 106 (12.2) |
| | Liver disease | 230,243 | 284 (123) | 1,069 (464) | 97 (42.1) | 173,423 | 208 (120) | 547 (315) | 75 (43.2) | 56,820 | 76 (134) | 522 (919) | 22 (38.7) |
| | CKD | 2,934,107 | 4,467 (152) | 25,816 (880) | 526 (17.9) | 978,886 | 1,179 (120) | 5,164 (528) | 200 (20.4) | 1,955,221 | 3,288 (168) | 20,652 (1,056) | 326 (16.7) |
| | Major surgery[3] | 3,995,870 | 5,407 (135) | 24,164 (605) | 763 (19.1) | 2,773,174 | 2,735 (98.6) | 9,898 (357) | 440 (15.9) | 1,222,696 | 2,672 (219) | 14,266 (1,167) | 323 (26.4) |
| | Dementia | 524,293 | 1,127 (215) | 6,073 (1,158) | 45 (8.6) | 48,821 | 81 (166) | 415 (850) | 10 (20.5) | 475,472 | 1,046 (220) | 5,658 (1,190) | 35 (7.4) |
| Medications | Antiplatelet | 2,510,382 | 2,814 (112) | 36,415 (1,451) | 218 (8.7) | 1,000,010 | 747 (74.7) | 15,556 (1,556) | 65 (6.5) | 1,510,372 | 2,067 (137) | 20,859 (1,381) | 153 (10.1) |
| | BP lowering | 8,589,860 | 7,969 (92.8) | 55,462 (646) | 777 (9.0) | 4,286,561 | 2,779 (64.8) | 20,579 (480) | 305 (7.1) | 4,303,299 | 5,190 (121) | 34,883 (811) | 472 (11.0) |
| | Lipid lowering | 6,808,408 | 5,796 (85.1) | 47,859 (703) | 564 (8.3) | 3,212,600 | 1,878 (58.5) | 19,543 (608) | 194 (6.0) | 3,595,808 | 3,918 (109) | 28,316 (787) | 370 (10.3) |
| | Anticoagulant | 1,338,585 | 2,278 (170) | 12,758 (953) | 205 (15.3) | 381,588 | 1,144 (300) | 2,773 (727) | 57 (14.9) | 956,997 | 1,134 (118) | 9,985 (1043) | 148 (15.5) |
| | Oral contraceptive | 622,529 | 138 (22.2) | 42 (6.7) | 8 (1.3) | 622,529 | 138 (22.2) | 42 (6.7) | 8 (1.3) | | | | |
| | HRT | 540,722 | 213 (39.4) | 516 (95.4) | 16 (3.0) | 501,957 | 185 (36.9) | 368 (73.3) | 14 (2.8) | 38,765 | 28 (72.2) | 148 (382) | <5 (5.2) |
| Number of diagnoses | 0 | 36,455,307 | 12,200 (33.5) | 38,617 (106) | 980 (2.7) | 31,819,272 | 7,612 (23.9) | 18,316 (57.6) | 643 (2.0) | 4,636,035 | 4,588 (99.0) | 20,301 (438) | 337 (7.3) |
| | 1 - 5 | 9,573,068 | 8,422 (88.0) | 46,012 (481) | 925 (9.7) | 6,872,479 | 4,137 (60.2) | 19,183 (279) | 515 (7.5) | 2,700,589 | 4,285 (159) | 26,829 (993) | 410 (15.2) |
| | 6+ | 134,567 | 281 (209) | 2,622 (1948) | 21 (15.6) | 52,420 | 86 (164) | 784 (1496) | 9 (17.2) | 82,147 | 195 (237) | 1,838 (2,237) | 12 (14.6) |
| Number of medications | 0 | 22,970,920 | 3,679 (16.0) | 9,629 (41.9) | 321 (1.4) | 22,122,043 | 3,143 (14.2) | 7,258 (32.8) | 290 (1.3) | 848,877 | 536 (63.1) | 2,371 (279) | 31 (3.7) |
| | 1 - 5 | 20,875,516 | 12,872 (61.7) | 56,255 (269) | 1,210 (5.8) | 15,618,756 | 7,203 (46.1) | 25,416 (163) | 713 (4.6) | 5,256,760 | 5,669 (108) | 30,839 (587) | 497 (9.5) |
| | 6+ | 2,316,506 | 4,352 (188) | 21,367 (922) | 395 (17.1) | 1,003,372 | 1,489 (148) | 5,609 (559) | 164 (16.3) | 1,313,134 | 2,863 (218) | 15,758 (1,200) | 231 (17.6) |

[1] Index of Multiple Deprivation deciles where 10 indicates least deprived and 1 indicates most deprived; [2] After 31/12/2019 and prior to 08/12/2020; [3] In the last year. BP: blood pressure, DCT: deep vein thrombosis; PE: pulmonary embolism; COPD: chronic obstructive respiratory disease; CKD: chronic kidney disease; BP: blood pressure; HRT: hormone replacement therapy; TCP: thrombocytopenia

**S1 Table (b). Numbers and incidence rates pre-, post-first ChAdOx1-S. Incidence rate per 100,000 person years. Disclosure control prevents presentation of n>5**

| | | Pre vaccination or unvaccinated | | Post vaccination ≤28 days | | Post vaccination >28 days | |
|---|---|---|---|---|---|---|---|
| **Outcome** | **Age** | **N (events)** | **Incidence rate (95% CI)** | **N (events)** | **Incidence rate (95% CI)** | **N (events)** | **Incidence rate (95% CI)** |
| **All venous** | All | 14,769 | 139.8 (137.6–142.1) | 2,006 | 293.6 (281.0–306.7) | 995 | 359.3 (337.6–382.3) |
| | <70 | 9,892 | 103.5 (101.5–105.6) | 902 | 206.8 (193.8–220.8) | 234 | 221.7 (195.0–252.0) |
| | ≥70 | 4877 | 483.8 (470.4–497.5) | 1,104 | 446.6 (421.0–473.7) | 761 | 444.0 (413.5–476.6) |
| Intracranial venous thrombosis | All | 207 | 1.96 (1.71–2.25) | 27 | 3.95 (2.71–5.76) | 9 | 3.25 (1.69–6.24) |
| | <70 | 188 | 1.97 (1.71–2.27) | 22 | 5.04 (3.32–7.66) | <10 | 4.74 (1.97–11.38) |
| | ≥70 | 19 | 1.88 (1.20–2.95) | 5 | 2.02 (0.84–4.86) | <5 | 2.33 (0.88–6.21) |
| Portal vein thrombosis | All | 121 | 1.15 (0.96–1.37) | 14 | 2.05 (1.21–3.46) | 9 | 3.25 (1.69–6.24) |
| | <70 | 98 | 1.03 (0.84–1.25) | 9 | 2.06 (1.07–3.96) | <5 | 1.89 (0.47–7.57) |
| | ≥70 | 23 | 2.28 (1.52–3.43) | 5 | 2.02 (0.84–4.86) | <10 | 4.08 (1.95–8.56) |
| Pulmonary embolism | All | 8,473 | 80.2 (78.5–81.9) | 1,203 | 176.0 (166.4–186.3) | 587 | 211.9 (195.4–229.7) |
| | <70 | 5,399 | 56.5 (55.0–58.0) | 515 | 118.1 (108.3–128.7) | 134 | 126.9 (107.2–150.4) |
| | ≥70 | 3,074 | 304.9 (294.3–315.9) | 688 | 278.2 (258.2–299.8) | 453 | 264.2 (240.9–289.7) |
| Deep vein thrombosis | All | 5,664 | 53.6 (52.2–55.0) | 739 | 108.1 (100.6–116.2) | 374 | 135.0 (122.0–149.4) |
| | <70 | 3,971 | 41.6 (40.3–42.9) | 345 | 79.1 (71.2–87.9) | 90 | 85.2 (69.3–104.8) |
| | ≥70 | 1,693 | 167.9 (160.1–176.1) | 394 | 159.3 (144.3–175.8) | 284 | 165.6 (147.4–186.0) |
| Other | All | 459 | 4.35 (3.97–4.76) | 51 | 7.46 (5.67–9.82) | 30 | 10.8 (7.6–15.5) |
| | <70 | 359 | 3.76 (3.39–4.17) | 34 | 7.79 (5.57–10.91) | 9 | 8.52 (4.43–16.38) |
| | ≥70 | 100 | 9.92 (8.15–12.06) | 17 | 6.87 (4.27–11.05) | 21 | 12.2 (8.0–18.8) |
| **All arterial** | All | 57,602 | 545.6 (541.1–550.0) | 8,256 | 1,210.6 (1184.8–1237.0) | 4,634 | 1,677.8 (1630.2–1726.8) |
| | <70 | 31,396 | 328.7 (325.1–332.4) | 2,993 | 687.3 (663.1–712.4) | 755 | 716.3 (667.0–769.2) |
| | ≥70 | 26,206 | 2,603.7 (2572.4–2635.5) | 5,263 | 2,135.3 (2078.3–2193.7) | 3,879 | 2,271.2 (2200.9–2343.8) |
| Myocardial infarction | All | 28,134 | 266.4 (263.3–269.5) | 3,814 | 558.5 (541.1–576.5) | 2,050 | 740.7 (709.4–773.5) |
| | <70 | 17,341 | 181.5 (178.8–184.2) | 1,628 | 373.5 (355.8–392.1) | 363 | 344.1 (310.4–381.3) |
| | ≥70 | 10,793 | 1,071.0 (1051.0–1091.4) | 2,186 | 885.0 (848.6–922.9) | 1,687 | 985.1 (939.2–1033.3) |
| Ischaemic stroke | All | 28,639 | 271.2 (268.1–274.3) | 4,334 | 634.8 (616.1–654.0) | 2,539 | 917.9 (882.9–954.3) |
| (ischaemic, unknown, spinal) | <70 | 13,505 | 141.4 (139.0–143.8) | 1,309 | 300.3 (284.5–317.0) | 377 | 357.3 (323.0–395.3) |
| | ≥70 | 15,134 | 1,502.4 (1478.7–1526.6) | 3,025 | 1,225.4 (1182.5–1269.9) | 2,162 | 1,263.5 (1211.3–1317.9) |
| Other arterial | All | 1,308 | 12.4 (11.7–13.1) | 189 | 27.6 (24.0–31.9) | 98 | 35.4 (29.0–43.1) |
| | <70 | 775 | 8.11 (7.56–8.70) | 82 | 18.8 (15.1–23.3) | 22 | 20.8 (13.7–31.6) |
| | ≥70 | 533 | 52.9 (48.6–57.5) | 107 | 43.3 (35.8–52.3) | 76 | 44.3 (35.4–55.5) |
| **Haematological** | | | | | | | |
| Disseminated intravascular coagulation | All | 13 | 0.12 (0.07–0.21) | <5 | 0.44 (0.14–1.36) | <5 | - |
| | <70 | 5 | 0.05 (0.02–0.13) | <5 | 0.46 (0.11–1.83) | <5 | - |
| | ≥70 | 8 | 0.79 (0.40–1.59) | <5 | 0.40 (0.06–2.87) | <5 | - |
| Thrombotic thrombocytopenic purpura | All | 70 | 0.66 (0.52–0.84) | <5 | 0.59 (0.22–1.56) | <5 | 0.72 (0.18–2.89) |
| | <70 | 64 | 0.67 (0.52–0.86) | <5 | 0.69 (0.22–2.13) | <5 | 1.89 (0.47–7.57) |
| | ≥70 | 6 | 0.59 (0.27–1.32) | <5 | 0.40 (0.06–2.87) | <5 | - |
| Any thrombocytopenia | All | 1,357 | 12.8 (12.2–13.5) | 200 | 29.3 (25.5–33.6) | 89 | 32.1 (26.1–39.5) |
| | <70 | 915 | 9.58 (8.98–10.22) | 124 | 28.4 (23.8–33.9) | 41 | 38.8 (28.6–52.7) |
| | ≥70 | 442 | 43.8 (39.9–48.1) | 76 | 30.7 (24.5–38.5) | 48 | 28.0 (21.1–37.1) |
| **Other** | | | | | | | |
| Haemorrhagic stroke | All | 3,747 | 35.5 (34.4–36.6) | 487 | 71.2 (65.2–77.9) | 289 | 104.3 (92.9–117.0) |
| (intracerebral or subarachnoid) | <70 | 2,028 | 21.2 (20.3–22.2) | 157 | 36.0 (30.8–42.1) | 45 | 42.6 (31.8–57.1) |
| | ≥70 | 1,719 | 170.5 (162.6–178.7) | 330 | 133.4 (119.8–148.6) | 244 | 142.2 (125.5–161.3) |
| Mesenteric thrombosis | All | 1,170 | 11.1 (10.5–11.7) | 171 | 25.0 (21.5–29.1) | 109 | 39.3 (32.6–47.5) |
| | <70 | 531 | 5.56 (5.10–6.05) | 56 | 12.8 (9.9–16.7) | 20 | 18.9 (12.2–29.4) |
| | ≥70 | 639 | 63.4 (58.6–68.5) | 115 | 46.5 (38.7–55.8) | 89 | 51.9 (42.1–63.9) |
| Lower limb fracture | All | 18,347 | 173.7 (171.2–176.3) | 2,657 | 389.0 (374.5–404.0) | 1,632 | 589.7 (561.7–619.0) |
| | <70 | 8,732 | 91.4 (89.5–93.3) | 539 | 123.6 (113.6–134.5) | 155 | 146.8 (125.4–171.9) |
| | ≥70 | 9,615 | 954.4 (935.5–973.7) | 2118 | 857.7 (821.9–895.0) | 1477 | 862.7 (819.8–907.8) |
| Death | All | 123,580 | 1,169.9 (1163.4–1176.4) | 16,192 | 2,368.5 (2332.3–2405.2) | 11,738 | 4,235.1 (4159.2–4312.4) |
| | <70 | 29,802 | 311.9 (308.4–315.5) | 1,940 | 444.7 (425.3–464.9) | 897 | 849.5 (795.7–906.9) |
| | ≥70 | 93,778 | 9,298.8 (9239.5–9358.5) | 14,252 | 5,761.2 (5667.4–5856.6) | 10,841 | 6,318.9 (6201.1–6439.0) |

**S1 Table (c). Numbers and incidence rates pre-, post-first BNT162b2. Incidence rate per 100,000 person years. Disclosure control prevents presentation of n>5**

| | Age | Pre vaccination or unvaccinated | | Post vaccination ≤28 days | | Post vaccination >28 days | |
|---|---|---|---|---|---|---|---|
| **Outcome** | **Age** | **N (events)** | **Incidence rate (95% CI)** | **N (events)** | **Incidence rate (95% CI)** | **N (events)** | **Incidence rate (95% CI)** |
| **All venous** | All | 14,769 | 139.8 (137.6–142.1) | 1,546 | 240.8 (229.1–253.1) | 1,587 | 277.1 (263.8–291.0) |
| | <70 | 9,892 | 103.5 (101.5–105.6) | 548 | 149.0 (137.0–162.0) | 259 | 106.3 (94.1–120.0) |
| | ≥70 | 4,877 | 483.8 (470.4–497.5) | 998 | 364.1 (342.2–387.5) | 1,328 | 403.6 (382.5–425.9) |
| Intracranial venous thrombosis | All | 207 | 1.96 (1.71–2.25) | 13 | 2.02 (1.18–3.49) | 6 | 1.05 (0.47–2.33) |
| | <70 | 188 | 1.97 (1.71–2.27) | 5 | 1.36 (0.57–3.26) | <5 | 1.23 (0.40–3.82) |
| | ≥70 | 19 | 1.88 (1.20–2.95) | 8 | 2.92 (1.46–5.83) | <5 | 0.91 (0.29–2.83) |
| Portal vein thrombosis | All | 121 | 1.15 (0.96–1.37) | 5 | 0.78 (0.32–1.87) | 12 | 2.09 (1.19–3.69) |
| | <70 | 98 | 1.03 (0.84–1.25) | <5 | 0.54 (0.14–2.17) | 6 | 2.46 (1.11–5.48) |
| | ≥70 | 23 | 2.28 (1.52–3.43) | <5 | 1.09 (0.35–3.39) | 6 | 1.82 (0.82–4.06) |
| Pulmonary embolism | All | 8,473 | 80.2 (78.5–81.9) | 928 | 144.5 (135.5–154.1) | 955 | 166.7 (156.5–177.6) |
| | <70 | 5,399 | 56.5 (55.0–58.0) | 306 | 83.2 (74.3–93.0) | 141 | 57.8 (49.0–68.2) |
| | ≥70 | 3,074 | 304.9 (294.3–315.9) | 622 | 226.9 (209.8–245.5) | 814 | 247.3 (230.9–264.9) |
| Deep vein thrombosis | All | 5,664 | 53.6 (52.2–55.0) | 555 | 86.4 (79.5–93.9) | 590 | 103.0 (95.0–111.6) |
| | <70 | 3,971 | 41.6 (40.3–42.9) | 213 | 57.9 (50.6–66.2) | 103 | 42.3 (34.8–51.3) |
| | ≥70 | 1,693 | 167.9 (160.1–176.1) | 342 | 124.8 (112.2–138.7) | 487 | 147.9 (135.4–161.7) |
| Other | All | 459 | 4.35 (3.97–4.76) | 55 | 8.56 (6.58–11.15) | 39 | 6.81 (4.97–9.31) |
| | <70 | 359 | 3.76 (3.39–4.17) | 28 | 7.61 (5.25–11.02) | 13 | 5.33 (3.10–9.18) |
| | ≥70 | 100 | 9.92 (8.15–12.06) | 27 | 9.85 (6.75–14.36) | 26 | 7.90 (5.38–11.60) |
| **All arterial** | All | 57,602 | 545.6 (541.1–550.0) | 7,960 | 1,241.8 (1214.9–1269.4) | 8,799 | 1,539.7 (1507.8–1572.2) |
| | <70 | 31,396 | 328.7 (325.1–332.4) | 2,263 | 615.8 (591.0–641.7) | 876 | 359.6 (336.6–384.2) |
| | ≥70 | 26,206 | 2,603.7 (2572.4–2635.5) | 5,697 | 2,082.9 (2029.5–2137.7) | 7,923 | 2,416.4 (2363.8–2470.2) |
| Myocardial infarction | All | 28,134 | 266.4 (263.3–269.5) | 3,722 | 580.1 (561.8–599.0) | 4,023 | 702.9 (681.5–724.9) |
| | <70 | 17,341 | 181.5 (178.8–184.2) | 1,285 | 349.5 (330.9–369.1) | 489 | 200.7 (183.6–219.3) |
| | ≥70 | 10,793 | 1,071.0 (1051.0–1091.4) | 2,437 | 889.8 (855.1–925.8) | 3,534 | 1,075.2 (1040.3–1111.2) |
| Ischaemic stroke (ischaemic, unknown, spinal) | All | 28,639 | 271.2 (268.1–274.3) | 4,143 | 645.7 (626.4–665.7) | 4,702 | 821.8 (798.6–845.6) |
| | <70 | 13,505 | 141.4 (139.0–143.8) | 949 | 258.0 (242.1–275.0) | 369 | 151.4 (136.7–167.7) |
| | ≥70 | 15,134 | 1,502.4 (1478.7–1526.6) | 3,194 | 1,166.5 (1126.7–1207.7) | 4,333 | 1,319.1 (1280.4–1359.0) |
| Other arterial | All | 1,308 | 12.4 (11.7–13.1) | 156 | 24.3 (20.8–28.4) | 167 | 29.1 (25.0–33.9) |
| | <70 | 775 | 8.11 (7.56–8.70) | 46 | 12.5 (9.4–16.7) | 26 | 10.7 (7.3–15.7) |
| | ≥70 | 533 | 52.9 (48.6–57.5) | 110 | 40.1 (33.3–48.4) | 141 | 42.8 (36.3–50.5) |
| **Haematological** | | | | | | | |
| Disseminated intravascular coagulation | All | 13 | 0.12 (0.07–0.21) | <5 | 0.31 (0.08–1.25) | <5 | 0.35 (0.09–1.40) |
| | <70 | 5 | 0.05 (0.02–0.13) | <5 | 0.27 (0.04–1.93) | <5 | - |
| | ≥70 | 8 | 0.79 (0.40–1.59) | <5 | 0.36 (0.05–2.59) | <5 | 0.61 (0.15–2.43) |
| Thrombotic thrombocytopenic purpura | All | 70 | 0.66 (0.52–0.84) | <5 | 0.62 (0.23–1.66) | <5 | 0.35 (0.09–1.40) |
| | <70 | 64 | 0.67 (0.52–0.86) | <5 | 0.82 (0.26–2.53) | <5 | 0.82 (0.21–3.28) |
| | ≥70 | 6 | 0.59 (0.27–1.32) | <5 | 0.36 (0.05–2.59) | <5 | - |
| Any thrombocytopenia | All | 1,357 | 12.8 (12.2–13.5) | 136 | 21.2 (17.9–25.1) | 144 | 25.1 (21.3–29.6) |
| | <70 | 915 | 9.58 (8.98–10.22) | 55 | 14.9 (11.5–19.5) | 32 | 13.1 (9.3–18.6) |
| | ≥70 | 442 | 43.8 (39.9–48.1) | 81 | 29.5 (23.8–36.7) | 112 | 34.0 (28.3–40.9) |
| **Other** | | | | | | | |
| Haemorrhagic stroke (intracerebral or subarachnoid) | All | 3,747 | 35.5 (34.4–36.6) | 440 | 68.5 (62.4–75.2) | 579 | 101.0 (93.1–109.6) |
| | <70 | 2,028 | 21.2 (20.3–22.2) | 93 | 25.3 (20.6–31.0) | 55 | 22.6 (17.3–29.4) |
| | ≥70 | 1,719 | 170.5 (162.6–178.7) | 347 | 126.6 (113.9–140.6) | 524 | 159.2 (146.1–173.4) |
| Mesenteric thrombosis | All | 1,170 | 11.1 (10.5–11.7) | 161 | 25.1 (21.5–29.3) | 217 | 37.9 (33.2–43.3) |
| | <70 | 531 | 5.56 (5.10–6.05) | 28 | 7.61 (5.25–11.02) | 18 | 7.38 (4.65–11.72) |
| | ≥70 | 639 | 63.4 (58.6–68.5) | 133 | 48.5 (40.9–57.5) | 199 | 60.4 (52.6–69.5) |
| Lower limb fracture | All | 18,347 | 173.7 (171.2–176.3) | 2,573 | 400.8 (385.6–416.6) | 3,176 | 554.7 (535.8–574.4) |
| | <70 | 8732 | 91.4 (89.5–93.3) | 443 | 120.4 (109.7–132.1) | 228 | 93.5 (82.2–106.5) |
| | ≥70 | 9615 | 954.4 (935.5–973.7) | 2,130 | 777.3 (745.0–811.0) | 2,948 | 896.6 (864.9–929.6) |
| Death | All | 123,580 | 1,169.9 (1163.4–1176.4) | 9,117 | 1,419.6 (1390.8–1449.0) | 13,935 | 2,431.7 (2391.7–2472.4) |
| | <70 | 29,802 | 311.9 (308.4–315.5) | 775 | 210.6 (196.3–225.9) | 531 | 217.8 (200.0–237.1) |
| | ≥70 | 93,778 | 9,298.8 (9239.5–9358.5) | 8,342 | 3,042.5 (2977.9–3108.5) | 13,404 | 4,071.0 (4002.7–4140.5) |

**S1 Table (d): Hazard ratios (95% CIs) for thrombotic and other outcomes 1-28 and >28 days post-vaccination with ChAdOx1-S vaccine, compared with pre-vaccination rates. All analyses were stratified on geographical region.**

| Outcome | Age | Sex | 1-28 days post-vaccination | | | >28 days post vaccination | | |
|---|---|---|---|---|---|---|---|---|
| | | | Unadjusted[1] | Age and sex adjusted[2] | Fully adjusted[3] | Unadjusted[1] | Age and sex adjusted[2] | Fully adjusted[3] |
| **All venous events** | <70 | Overall | 2.33 (2.16–2.51) | 1.32 (1.22–1.43) | 0.97 (0.90–1.05) | 2.74 (2.39–3.14) | 1.60 (1.38–1.84) | 0.94 (0.81–1.08) |
| | | Men | 2.68 (2.41–2.97) | 1.33 (1.19–1.49) | 1.04 (0.93–1.16) | 3.13 (2.54–3.84) | 1.52 (1.23–1.89) | 0.97 (0.78–1.20) |
| | | Women | 2.08 (1.87–2.31) | 1.31 (1.17–1.47) | 0.96 (0.85–1.07) | 2.56 (2.13–3.07) | 1.67 (1.38–2.02) | 1.02 (0.84–1.23) |
| | ≥70 | Overall | 0.65 (0.59–0.72) | 0.64 (0.59–0.70) | 0.58 (0.53–0.63) | 0.67 (0.59–0.76) | 0.58 (0.52–0.65) | 0.49 (0.44–0.55) |
| | | Men | 0.65 (0.56–0.76) | 0.65 (0.56–0.74) | 0.59 (0.51–0.68) | 0.70 (0.58–0.84) | 0.63 (0.53–0.75) | 0.55 (0.46–0.65) |
| | | Women | 0.65 (0.58–0.74) | 0.64 (0.56–0.71) | 0.57 (0.50–0.64) | 0.64 (0.54–0.76) | 0.54 (0.47–0.64) | 0.45 (0.39–0.53) |
| Intracranial venous thrombosis | <70 | Overall | 2.81 (1.70–4.64) | 2.99 (1.75–5.09) | 2.27 (1.33–3.88) | 2.37 (0.91–6.12) | 2.47 (0.95–6.42) | 1.72 (0.66–4.49) |
| | | Men | 2.72 (1.15–6.39) | 2.58 (0.99–6.74) | 2.06 (0.79–5.37) | 3.43 (0.74–15.87) | 3.37 (0.71–15.90) | 2.40 (0.53–10.86) |
| | | Women | 2.69 (1.45–5.00) | 3.27 (1.74–6.17) | 2.24 (1.17–4.31) | 1.75 (0.52–5.93) | 2.14 (0.63–7.22) | 1.47 (0.43–5.04) |
| | ≥70 | Overall | 0.62 (0.17–2.23) | 0.65 (0.19–2.21) | 0.67 (0.20–2.18) | 1.35 (0.13–13.46) | 1.13 (0.14–9.20) | 1.11 (0.14–9.02) |
| | | Men | 10.8 (3.3–35.8) | 8.99 (2.75–29.42) | 10.1 (2.7–38.5) | - | - | - |
| | | Women | 0.27 (0.05–1.42) | 0.29 (0.06–1.43) | 0.28 (0.06–1.34) | 0.87 (0.10–7.32) | 0.82 (0.11–6.24) | 0.76 (0.10–5.70) |
| Portal vein thrombosis | <70 | Overall | 2.49 (1.20–5.14) | 1.56 (0.72–3.40) | 1.00 (0.24–4.15) | 2.76 (0.73–10.51) | 1.99 (0.53–7.52) | 1.00 (0.06–18.18) |
| | | Men | 3.38 (1.40–8.17) | 1.95 (0.76–5.02) | 1.22 (0.48–3.08) | 3.55 (0.52–24.13) | 2.16 (0.32–14.52) | 0.98 (0.14–6.75) |
| | | Women | 1.74 (0.48–6.26) | 1.06 (0.28–4.07) | 0.72 (0.18–2.86) | 2.39 (0.37–15.53) | 1.57 (0.24–10.12) | 0.86 (0.12–6.28) |
| | ≥70 | Overall | 0.66 (0.11–4.06) | 0.67 (0.10–4.24) | 0.61 (0.10–3.71) | 2.72 (0.49–15.02) | 3.00 (0.48–18.99) | 2.61 (0.41–16.52) |
| | | Men | 0.43 (0.09–2.09) | 0.42 (0.08–2.15) | 0.37 (0.07–1.90) | 8.60 (2.33–31.70) | 7.99 (1.93–33.03) | 7.99 (2.03–31.38) |
| | | Women | 0.92 (0.06–13.23) | 0.92 (0.06–13.99) | 0.82 (0.06–11.05) | 0.87 (0.12–6.02) | 0.95 (0.12–7.39) | 0.86 (0.11–6.92) |
| Pulmonary embolism | <70 | Overall | 2.54 (2.30–2.80) | 1.28 (1.15–1.43) | 0.95 (0.85–1.05) | 3.12 (2.61–3.73) | 1.63 (1.34–1.96) | 0.95 (0.79–1.15) |
| | | Men | 2.83 (2.46–3.24) | 1.27 (1.09–1.48) | 1.01 (0.87–1.17) | 3.32 (2.52–4.38) | 1.43 (1.07–1.91) | 0.92 (0.69–1.23) |
| | | Women | 2.35 (2.04–2.70) | 1.30 (1.12–1.52) | 0.95 (0.82–1.10) | 3.09 (2.44–3.92) | 1.81 (1.41–2.32) | 1.09 (0.85–1.41) |
| | ≥70 | Overall | 0.62 (0.55–0.70) | 0.60 (0.54–0.68) | 0.54 (0.48–0.61) | 0.61 (0.52–0.72) | 0.54 (0.46–0.62) | 0.45 (0.39–0.53) |
| | | Men | 0.63 (0.52–0.75) | 0.62 (0.52–0.74) | 0.57 (0.47–0.67) | 0.63 (0.50–0.79) | 0.57 (0.46–0.70) | 0.49 (0.40–0.62) |
| | | Women | 0.61 (0.52–0.72) | 0.59 (0.51–0.69) | 0.53 (0.45–0.61) | 0.60 (0.48–0.75) | 0.51 (0.42–0.63) | 0.43 (0.35–0.53) |
| Deep vein thrombosis | <70 | Overall | 2.08 (1.85–2.34) | 1.34 (1.18–1.52) | 0.99 (0.87–1.12) | 2.37 (1.90–2.96) | 1.58 (1.25–1.99) | 0.97 (0.77–1.22) |
| | | Men | 2.61 (2.22–3.07) | 1.48 (1.25–1.76) | 1.15 (0.97–1.36) | 2.85 (2.04–3.98) | 1.62 (1.14–2.29) | 1.06 (0.75–1.50) |
| | | Women | 1.67 (1.40–2.00) | 1.21 (1.00–1.46) | 0.90 (0.75–1.09) | 2.12 (1.57–2.86) | 1.57 (1.15–2.13) | 1.02 (0.75–1.40) |
| | ≥70 | Overall | 0.73 (0.62–0.86) | 0.71 (0.61–0.83) | 0.63 (0.54–0.74) | 0.71 (0.57–0.88) | 0.62 (0.51–0.75) | 0.51 (0.42–0.62) |
| | | Men | 0.72 (0.56–0.94) | 0.71 (0.55–0.91) | 0.63 (0.49–0.81) | 0.77 (0.56–1.06) | 0.70 (0.51–0.95) | 0.58 (0.42–0.79) |
| | | Women | 0.73 (0.59–0.90) | 0.72 (0.59–0.87) | 0.64 (0.53–0.77) | 0.67 (0.51–0.88) | 0.57 (0.45–0.73) | 0.47 (0.37–0.60) |
| Other | <70 | Overall | 2.48 (1.70–3.62) | 1.60 (1.06–2.40) | 1.02 (0.69–1.52) | 3.04 (1.54–6.00) | 2.04 (1.01–4.09) | 0.94 (0.47–1.91) |
| | | Men | 2.61 (1.51–4.53) | 1.45 (0.79–2.64) | 1.02 (0.57–1.83) | 3.17 (1.13–8.90) | 1.74 (0.60–5.05) | 0.89 (0.31–2.58) |
| | | Women | 2.38 (1.42–4.00) | 1.74 (1.01–2.98) | 1.12 (0.65–1.91) | 3.15 (1.29–7.73) | 2.40 (0.97–5.96) | 1.26 (0.50–3.17) |
| | ≥70 | Overall | 0.46 (0.21–1.01) | 0.46 (0.21–0.99) | 0.43 (0.20–0.92) | 1.13 (0.50–2.54) | 1.03 (0.48–2.21) | 0.93 (0.42–2.03) |
| | | Men | 0.38 (0.11–1.30) | 0.39 (0.12–1.31) | 0.33 (0.10–1.08) | 1.10 (0.36–3.39) | 1.02 (0.34–3.04) | 0.79 (0.26–2.36) |
| | | Women | 0.52 (0.19–1.44) | 0.51 (0.20–1.36) | 0.50 (0.19–1.32) | 1.16 (0.37–3.63) | 1.03 (0.36–2.98) | 0.98 (0.33–2.94) |
| **All arterial events** | <70 | Overall | 3.02 (2.90–3.14) | 1.25 (1.20–1.31) | 0.90 (0.86–0.95) | 3.55 (3.29–3.83) | 1.57 (1.45–1.70) | 0.91 (0.84–1.00) |
| | | Men | 3.42 (3.26–3.60) | 1.26 (1.20–1.34) | 0.91 (0.86–0.96) | 4.70 (4.27–5.17) | 1.66 (1.50–1.84) | 0.96 (0.86–1.07) |
| | | Women | 2.96 (2.76–3.18) | 1.24 (1.14–1.34) | 0.93 (0.86–1.00) | 3.30 (2.90–3.75) | 1.43 (1.25–1.64) | 0.88 (0.77–1.02) |
| | ≥70 | Overall | 0.89 (0.85–0.93) | 0.84 (0.80–0.87) | 0.76 (0.73–0.79) | 1.03 (0.97–1.10) | 0.83 (0.78–0.87) | 0.72 (0.68–0.77) |
| | | Men | 0.90 (0.85–0.96) | 0.87 (0.82–0.92) | 0.78 (0.74–0.83) | 1.06 (0.97–1.16) | 0.89 (0.82–0.96) | 0.78 (0.71–0.85) |
| | | Women | 0.88 (0.82–0.94) | 0.80 (0.76–0.85) | 0.73 (0.69–0.78) | 1.02 (0.93–1.12) | 0.77 (0.71–0.83) | 0.68 (0.62–0.74) |
| Myocardial infarction | <70 | Overall | 2.85 (2.70–3.01) | 1.24 (1.17–1.32) | 0.88 (0.83–0.94) | 2.93 (2.63–3.26) | 1.40 (1.24–1.56) | 0.83 (0.73–0.93) |
| | | Men | 3.23 (3.02–3.44) | 1.25 (1.17–1.35) | 0.89 (0.83–0.95) | 3.52 (3.08–4.03) | 1.33 (1.15–1.53) | 0.78 (0.68–0.90) |
| | | Women | 2.93 (2.63–3.26) | 1.22 (1.09–1.38) | 0.92 (0.82–1.04) | 3.56 (2.95–4.29) | 1.58 (1.29–1.92) | 1.01 (0.82–1.24) |
| | ≥70 | Overall | 0.86 (0.80–0.92) | 0.83 (0.78–0.88) | 0.76 (0.71–0.81) | 0.97 (0.89–1.07) | 0.83 (0.76–0.90) | 0.74 (0.68–0.81) |
| | | Men | 0.88 (0.80–0.96) | 0.85 (0.78–0.93) | 0.77 (0.71–0.84) | 0.96 (0.84–1.09) | 0.84 (0.74–0.94) | 0.73 (0.64–0.82) |
| | | Women | 0.85 (0.76–0.94) | 0.80 (0.72–0.88) | 0.76 (0.69–0.84) | 1.03 (0.90–1.19) | 0.82 (0.72–0.93) | 0.78 (0.68–0.88) |

| Outcome | Age | Sex | 1-28 days post-vaccination | | | >28 days post vaccination | | |
|---|---|---|---|---|---|---|---|---|
| | | | Unadjusted[1] | Age and sex adjusted[2] | Fully adjusted[3] | Unadjusted[1] | Age and sex adjusted[2] | Fully adjusted[3] |
| Ischaemic stroke[4] | <70 | Overall | 3.21 (3.02–3.41) | 1.25 (1.17–1.34) | 0.90 (0.84–0.96) | 4.36 (3.91–4.86) | 1.76 (1.56–1.98) | 0.94 (0.84–1.07) |
| | | Men | 3.73 (3.44–4.05) | 1.28 (1.17–1.40) | 0.91 (0.83–0.99) | 6.58 (5.73–7.57) | 2.14 (1.84–2.49) | 1.12 (0.96–1.31) |
| | | Women | 2.91 (2.64–3.20) | 1.21 (1.09–1.35) | 0.90 (0.81–1.00) | 3.10 (2.59–3.71) | 1.32 (1.09–1.59) | 0.77 (0.63–0.94) |
| | ≥70 | Overall | 0.92 (0.87–0.98) | 0.85 (0.81–0.90) | 0.77 (0.73–0.82) | 1.08 (0.99–1.18) | 0.83 (0.77–0.90) | 0.72 (0.67–0.78) |
| | | Men | 0.94 (0.85–1.03) | 0.89 (0.82–0.96) | 0.81 (0.74–0.88) | 1.14 (1.01–1.30) | 0.93 (0.83–1.04) | 0.82 (0.73–0.92) |
| | | Women | 0.92 (0.84–1.00) | 0.82 (0.77–0.89) | 0.75 (0.69–0.80) | 1.04 (0.92–1.17) | 0.76 (0.68–0.84) | 0.66 (0.59–0.73) |
| Other arterial | <70 | Overall | 3.18 (2.48–4.07) | 1.24 (0.93–1.64) | 0.82 (0.63–1.07) | 4.20 (2.62–6.73) | 1.68 (0.99–2.86) | 0.77 (0.46–1.30) |
| | | Men | 3.17 (2.29–4.40) | 1.05 (0.73–1.51) | 0.75 (0.53–1.07) | 5.56 (3.07–10.06) | 1.66 (0.86–3.20) | 0.89 (0.47–1.69) |
| | | Women | 3.67 (2.51–5.37) | 1.59 (1.03–2.46) | 1.04 (0.68–1.59) | 3.60 (1.64–7.91) | 1.74 (0.73–4.18) | 0.83 (0.35–2.01) |
| | ≥70 | Overall | 0.44 (0.33–0.59) | 0.45 (0.34–0.59) | 0.41 (0.31–0.53) | 0.55 (0.38–0.79) | 0.49 (0.35–0.70) | 0.44 (0.31–0.63) |
| | | Men | 0.46 (0.31–0.67) | 0.45 (0.31–0.66) | 0.42 (0.29–0.61) | 0.61 (0.35–1.05) | 0.56 (0.33–0.95) | 0.51 (0.30–0.87) |
| | | Women | 0.43 (0.28–0.66) | 0.44 (0.30–0.67) | 0.39 (0.26–0.59) | 0.51 (0.30–0.86) | 0.44 (0.27–0.71) | 0.38 (0.23–0.62) |
| **Haematological events** | | | | | | | | |
| Any thrombocytopenia | <70 | Overall | 4.85 (3.87–6.07) | 3.00 (2.34–3.85) | 1.71 (1.35–2.16) | 7.48 (5.31–10.53) | 4.68 (3.24–6.77) | 1.69 (1.16–2.46) |
| | | Men | 6.47 (4.62–9.06) | 3.52 (2.42–5.13) | 1.85 (1.30–2.63) | 13.2 (8.1–21.4) | 6.84 (3.99–11.73) | 2.02 (1.18–3.44) |
| | | Women | 3.81 (2.80–5.17) | 2.65 (1.89–3.71) | 1.95 (1.40–2.72) | 4.59 (2.78–7.58) | 3.34 (1.98–5.64) | 2.08 (1.22–3.54) |
| | ≥70 | Overall | 0.92 (0.65–1.29) | 0.87 (0.63–1.22) | 0.79 (0.56–1.10) | 1.14 (0.66–1.95) | 0.99 (0.59–1.65) | 0.84 (0.50–1.41) |
| | | Men | 0.84 (0.53–1.32) | 0.79 (0.50–1.22) | 0.68 (0.43–1.06) | 1.37 (0.64–2.91) | 1.17 (0.57–2.38) | 0.92 (0.45–1.90) |
| | | Women | 1.04 (0.62–1.75) | 0.99 (0.61–1.63) | 0.93 (0.56–1.54) | 1.02 (0.46–2.24) | 0.88 (0.42–1.85) | 0.80 (0.38–1.70) |
| **Other events** | | | | | | | | |
| Haemorrhagic stroke[5] | <70 | Overall | 2.25 (1.88–2.69) | 1.05 (0.86–1.27) | 1.00 (0.73–1.37) | 2.87 (2.10–3.92) | 1.39 (1.00–1.93) | 1.00 (0.51–1.95) |
| | | Men | 2.68 (2.11–3.41) | 1.15 (0.89–1.49) | 1.01 (0.78–1.30) | 2.86 (1.74–4.71) | 1.21 (0.72–2.04) | 0.87 (0.52–1.46) |
| | | Women | 1.93 (1.48–2.52) | 0.93 (0.70–1.24) | 0.87 (0.66–1.16) | 2.94 (1.95–4.42) | 1.47 (0.95–2.27) | 1.23 (0.80–1.91) |
| | ≥70 | Overall | 0.83 (0.69–1.00) | 0.78 (0.66–0.92) | 0.73 (0.62–0.87) | 1.09 (0.84–1.40) | 0.84 (0.67–1.04) | 0.76 (0.61–0.95) |
| | | Men | 0.86 (0.65–1.14) | 0.82 (0.64–1.05) | 0.79 (0.62–1.01) | 1.34 (0.93–1.94) | 1.03 (0.75–1.43) | 0.97 (0.70–1.34) |
| | | Women | 0.81 (0.63–1.03) | 0.75 (0.60–0.94) | 0.70 (0.56–0.87) | 0.92 (0.65–1.30) | 0.71 (0.52–0.96) | 0.64 (0.47–0.86) |
| Mesenteric thrombosis | <70 | Overall | 3.25 (2.42–4.37) | 1.24 (0.89–1.73) | 0.84 (0.61–1.16) | 5.47 (3.33–8.99) | 2.16 (1.24–3.77) | 1.04 (0.60–1.80) |
| | | Men | 2.87 (1.72–4.77) | 0.98 (0.57–1.71) | 0.72 (0.43–1.22) | 4.34 (1.59–11.83) | 1.42 (0.48–4.25) | 0.75 (0.26–2.17) |
| | | Women | 3.39 (2.36–4.87) | 1.43 (0.94–2.17) | 1.01 (0.67–1.52) | 5.66 (3.21–9.99) | 2.59 (1.36–4.94) | 1.40 (0.73–2.69) |
| | ≥70 | Overall | 0.66 (0.49–0.89) | 0.60 (0.46–0.79) | 0.53 (0.41–0.70) | 0.95 (0.64–1.43) | 0.74 (0.52–1.05) | 0.62 (0.43–0.89) |
| | | Men | 0.65 (0.39–1.09) | 0.61 (0.38–0.98) | 0.52 (0.32–0.83) | 0.81 (0.44–1.49) | 0.69 (0.40–1.18) | 0.54 (0.31–0.94) |
| | | Women | 0.66 (0.46–0.95) | 0.60 (0.44–0.84) | 0.55 (0.40–0.77) | 1.00 (0.60–1.68) | 0.76 (0.48–1.20) | 0.67 (0.42–1.08) |
| Lower limb fracture | <70 | Overall | 1.71 (1.55–1.87) | 1.01 (0.91–1.11) | 0.85 (0.77–0.93) | 2.26 (1.91–2.67) | 1.35 (1.13–1.60) | 0.98 (0.83–1.17) |
| | | Men | 1.35 (1.15–1.58) | 0.98 (0.83–1.16) | 0.86 (0.73–1.02) | 2.22 (1.67–2.93) | 1.60 (1.20–2.14) | 1.24 (0.93–1.66) |
| | | Women | 1.91 (1.70–2.15) | 1.03 (0.91–1.16) | 0.88 (0.78–0.99) | 2.21 (1.80–2.72) | 1.25 (1.01–1.55) | 0.95 (0.77–1.18) |
| | ≥70 | Overall | 1.11 (1.02–1.21) | 0.94 (0.88–1.01) | 0.83 (0.78–0.89) | 1.56 (1.39–1.76) | 0.95 (0.87–1.04) | 0.78 (0.71–0.85) |
| | | Men | 1.06 (0.90–1.24) | 0.94 (0.83–1.08) | 0.84 (0.73–0.95) | 1.50 (1.19–1.89) | 0.97 (0.81–1.17) | 0.81 (0.67–0.97) |
| | | Women | 1.11 (1.01–1.22) | 0.95 (0.88–1.03) | 0.84 (0.77–0.91) | 1.51 (1.32–1.73) | 0.95 (0.85–1.06) | 0.78 (0.70–0.87) |
| Death | <70 | Overall | 1.96 (1.87–2.06) | 0.60 (0.57–0.63) | 0.37 (0.35–0.39) | 4.51 (4.20–4.85) | 1.41 (1.30–1.53) | 0.51 (0.47–0.56) |
| | | Men | 2.35 (2.20–2.51) | 0.63 (0.58–0.67) | 0.44 (0.41–0.47) | 6.47 (5.87–7.13) | 1.57 (1.40–1.75) | 0.75 (0.67–0.85) |
| | | Women | 1.69 (1.56–1.82) | 0.56 (0.52–0.61) | 0.36 (0.33–0.39) | 3.48 (3.13–3.89) | 1.21 (1.07–1.37) | 0.50 (0.44–0.57) |
| | ≥70 | Overall | 0.35 (0.34–0.36) | 0.34 (0.33–0.35) | 0.28 (0.28–0.29) | 0.49 (0.47–0.51) | 0.38 (0.37–0.39) | 0.27 (0.26–0.28) |
| | | Men | 0.31 (0.30–0.32) | 0.32 (0.31–0.33) | 0.26 (0.25–0.27) | 0.44 (0.42–0.47) | 0.37 (0.35–0.38) | 0.26 (0.24–0.27) |
| | | Women | 0.39 (0.37–0.40) | 0.36 (0.35–0.37) | 0.30 (0.29–0.31) | 0.54 (0.51–0.56) | 0.39 (0.38–0.41) | 0.29 (0.27–0.30) |

[1]Stratifed by region
[2]Adjusted for age, age$^2$, sex and stratified by region
[3]Adjusted for age, age$^2$, sex, ethnicity, deprivation, smoking, previous diagnosis of cancer, number of unique diseases in the last year, surgery in the last year, obesity, liver disease; additionally for women aged <70, history of depression and total number of types of medication (by BNF chapters); additionally for men aged <70, history of SARS-CoV2 infection and thrombocytopenia; and additionally for men and women aged ≥70, chronic kidney disease and dementia. Venous events adjusted for: anticoagulant medication, combined oral contraceptive medication, hormone replacement therapy medication, history of PE or DVT, and history of coronavirus infection. Arterial thromboses adjusted for: diabetes, hypertension, smoking, antiplatelet medication, blood pressure lowering medication, lipid lowering medication, anticoagulant medication, history of stroke, and history of MI. All analyses stratified by region.
[4]Ischaemic stroke: including ischaemic stroke, stroke of uncertain cause, and spinal stroke
[5]Hemorrhagic stroke: including intracranial hemorrhage and subarachnoid hemorrhage

**S1 Table (e): Hazard ratios (95% CIs) for thrombotic and other outcomes 1-28 and >28 days post-vaccination, compared with pre-vaccination rates. All analyses were stratified on geographical region BNT162b2 vaccine**

| **Outcome** | **Age** | **Sex** | **1-28 days post-vaccination** | | | **>28 days post vaccination** | | |
|---|---|---|---|---|---|---|---|---|
| | | | **Unadjusted[1]** | **Age sex adjusted[2]** | **Fully adjusted[3]** | **Unadjusted[1]** | **Age sex adjusted[2]** | **Fully adjusted[3]** |
| **All venous events** | <70 | Overall | 1.47 (1.35–1.61) | 1.01 (0.93–1.11) | 0.81 (0.74–0.88) | 1.14 (1.01–1.29) | 0.88 (0.77–1.00) | 0.73 (0.64–0.83) |
| | | Men | 1.98 (1.75–2.23) | 1.15 (1.01–1.30) | 0.92 (0.81–1.05) | 1.56 (1.28–1.89) | 1.02 (0.83–1.25) | 0.83 (0.68–1.02) |
| | | Women | 1.20 (1.06–1.37) | 0.91 (0.80–1.03) | 0.74 (0.65–0.84) | 1.01 (0.86–1.20) | 0.81 (0.69–0.96) | 0.70 (0.59–0.83) |
| | ≥70 | Overall | 0.65 (0.60–0.71) | 0.56 (0.52–0.61) | 0.57 (0.53–0.62) | 0.68 (0.62–0.75) | 0.50 (0.46–0.55) | 0.50 (0.45–0.55) |
| | | Men | 0.67 (0.59–0.76) | 0.59 (0.52–0.66) | 0.60 (0.53–0.67) | 0.75 (0.65–0.87) | 0.59 (0.51–0.68) | 0.58 (0.50–0.67) |
| | | Women | 0.64 (0.58–0.72) | 0.54 (0.49–0.60) | 0.56 (0.50–0.63) | 0.63 (0.56–0.72) | 0.45 (0.40–0.51) | 0.45 (0.40–0.52) |
| Intracranial venous thrombosis | <70 | Overall | 0.76 (0.31–1.85) | 0.72 (0.29–1.78) | 0.59 (0.24–1.45) | 0.65 (0.20–2.13) | 0.59 (0.18–1.93) | 0.51 (0.16–1.68) |
| | | Men | 1.60 (0.50–5.16) | 1.39 (0.43–4.49) | 1.09 (0.33–3.61) | 1.04 (0.14–7.77) | 0.92 (0.12–7.05) | 0.74 (0.10–5.62) |
| | | Women | 0.39 (0.10–1.60) | 0.44 (0.11–1.78) | 0.37 (0.09–1.52) | 0.48 (0.11–2.07) | 0.51 (0.12–2.20) | 0.45 (0.10–1.95) |
| | ≥70 | Overall | 1.65 (0.64–4.29) | 1.25 (0.48–3.26) | 1.43 (0.55–3.75) | 1.13 (0.13–9.75) | 0.71 (0.08–6.56) | 0.88 (0.10–7.52) |
| | | Men | - | - | - | - | - | - |
| | | Women | 2.01 (0.69–5.88) | 1.45 (0.49–4.26) | 1.57 (0.54–4.54) | 1.26 (0.13–11.94) | 0.73 (0.07–7.29) | 0.82 (0.09–7.61) |
| Portal vein thrombosis | <70 | Overall | 0.58 (0.15–2.30) | 0.44 (0.11–1.72) | 0.29 (0.07–1.17) | 3.06 (1.29–7.24) | 2.84 (1.19–6.74) | 2.19 (0.89–5.37) |
| | | Men | 1.28 (0.33–4.95) | 0.81 (0.21–3.09) | 0.51 (0.13–2.04) | - | - | - |
| | | Women | - | - | - | 5.30 (1.98–14.22) | 4.28 (1.55–11.78) | 3.95 (1.39–11.20) |
| | ≥70 | Overall | 0.49 (0.10–2.28) | 0.43 (0.10–1.87) | 0.42 (0.09–1.91) | 0.67 (0.23–1.97) | 0.54 (0.19–1.56) | 0.48 (0.17–1.38) |
| | | Men | 0.85 (0.17–4.38) | 0.55 (0.11–2.66) | 0.49 (0.10–2.46) | 1.26 (0.36–4.41) | 0.70 (0.23–2.17) | 0.48 (0.17–1.38) |
| | | Women | 0.22 (0.01–3.38) | 0.24 (0.01–3.80) | 0.26 (0.02–4.47) | 0.37 (0.09–1.52) | 0.44 (0.10–2.01) | 0.46 (0.11–2.04) |
| Pulmonary embolism | <70 | Overall | 1.52 (1.36–1.71) | 0.99 (0.87–1.11) | 0.78 (0.69–0.88) | 1.20 (1.02–1.43) | 0.89 (0.75–1.06) | 0.71 (0.60–0.85) |
| | | Men | 1.93 (1.63–2.28) | 1.05 (0.88–1.25) | 0.85 (0.71–1.01) | 1.56 (1.19–2.05) | 0.97 (0.74–1.29) | 0.77 (0.58–1.03) |
| | | Women | 1.32 (1.13–1.56) | 0.94 (0.79–1.10) | 0.75 (0.64–0.89) | 1.10 (0.88–1.37) | 0.84 (0.67–1.05) | 0.71 (0.57–0.89) |
| | ≥70 | Overall | 0.61 (0.55–0.68) | 0.53 (0.48–0.59) | 0.54 (0.49–0.60) | 0.61 (0.54–0.69) | 0.45 (0.40–0.51) | 0.45 (0.39–0.50) |
| | | Men | 0.64 (0.55–0.75) | 0.56 (0.48–0.65) | 0.57 (0.49–0.66) | 0.65 (0.54–0.77) | 0.50 (0.42–0.59) | 0.49 (0.41–0.59) |
| | | Women | 0.59 (0.52–0.68) | 0.51 (0.44–0.58) | 0.52 (0.45–0.60) | 0.58 (0.49–0.68) | 0.42 (0.36–0.49) | 0.42 (0.36–0.49) |
| Deep vein thrombosis | <70 | Overall | 1.38 (1.21–1.59) | 1.03 (0.89–1.18) | 0.82 (0.71–0.95) | 1.05 (0.86–1.28) | 0.85 (0.69–1.04) | 0.73 (0.59–0.89) |
| | | Men | 1.93 (1.60–2.33) | 1.23 (1.01–1.49) | 0.99 (0.81–1.20) | 1.55 (1.15–2.10) | 1.10 (0.81–1.48) | 0.94 (0.69–1.28) |
| | | Women | 1.08 (0.88–1.33) | 0.87 (0.71–1.08) | 0.72 (0.58–0.89) | 0.88 (0.67–1.15) | 0.74 (0.56–0.97) | 0.65 (0.50–0.86) |
| | ≥70 | Overall | 0.69 (0.60–0.79) | 0.58 (0.51–0.67) | 0.61 (0.53–0.70) | 0.81 (0.69–0.96) | 0.59 (0.50–0.70) | 0.61 (0.52–0.73) |
| | | Men | 0.66 (0.53–0.82) | 0.59 (0.48–0.73) | 0.61 (0.49–0.76) | 0.95 (0.74–1.23) | 0.78 (0.60–1.01) | 0.80 (0.61–1.05) |
| | | Women | 0.71 (0.59–0.85) | 0.59 (0.49–0.70) | 0.62 (0.52–0.74) | 0.72 (0.58–0.90) | 0.49 (0.40–0.61) | 0.52 (0.41–0.64) |
| Other | <70 | Overall | 2.16 (1.47–3.19) | 1.60 (1.07–2.37) | 1.18 (0.79–1.77) | 1.64 (0.93–2.90) | 1.34 (0.75–2.40) | 1.03 (0.57–1.87) |
| | | Men | 3.25 (1.93–5.50) | 2.03 (1.16–3.54) | 1.46 (0.83–2.57) | 2.14 (0.86–5.34) | 1.51 (0.59–3.86) | 1.03 (0.39–2.70) |
| | | Women | 1.48 (0.82–2.65) | 1.20 (0.67–2.16) | 0.98 (0.54–1.77) | 1.55 (0.75–3.21) | 1.31 (0.63–2.74) | 1.21 (0.57–2.55) |
| | ≥70 | Overall | 1.13 (0.66–1.94) | 0.97 (0.58–1.64) | 1.01 (0.60–1.72) | 0.86 (0.39–1.88) | 0.66 (0.31–1.41) | 0.67 (0.30–1.47) |
| | | Men | 1.50 (0.77–2.95) | 1.25 (0.65–2.42) | 1.27 (0.64–2.49) | 1.51 (0.50–4.51) | 1.09 (0.36–3.34) | 1.07 (0.33–3.44) |
| | | Women | 0.79 (0.31–2.04) | 0.72 (0.30–1.75) | 0.75 (0.30–1.85) | 0.42 (0.17–1.06) | 0.36 (0.15–0.88) | 0.36 (0.14–0.91) |
| **All arterial events** | <70 | Overall | 2.21 (2.12–2.31) | 1.31 (1.25–1.37) | 0.94 (0.90–0.99) | 1.45 (1.35–1.55) | 1.08 (1.01–1.16) | 0.88 (0.82–0.95) |
| | | Men | 3.06 (2.90–3.22) | 1.44 (1.36–1.52) | 0.99 (0.93–1.05) | 2.17 (1.99–2.38) | 1.19 (1.08–1.31) | 0.91 (0.82–1.00) |
| | | Women | 1.85 (1.71–1.99) | 1.11 (1.02–1.20) | 0.92 (0.84–0.99) | 1.44 (1.30–1.60) | 0.96 (0.86–1.07) | 0.91 (0.81–1.02) |
| | ≥70 | Overall | 0.90 (0.87–0.93) | 0.69 (0.66–0.71) | 0.72 (0.70–0.75) | 1.15 (1.10–1.20) | 0.69 (0.66–0.72) | 0.71 (0.68–0.74) |
| | | Men | 0.96 (0.91–1.00) | 0.75 (0.71–0.78) | 0.77 (0.73–0.81) | 1.17 (1.11–1.25) | 0.74 (0.70–0.79) | 0.74 (0.70–0.79) |
| | | Women | 0.84 (0.80–0.89) | 0.63 (0.60–0.66) | 0.68 (0.65–0.71) | 1.13 (1.06–1.20) | 0.64 (0.61–0.68) | 0.68 (0.64–0.73) |
| Myocardial infarction | <70 | Overall | 2.24 (2.12–2.37) | 1.39 (1.30–1.47) | 0.94 (0.88–1.00) | 1.42 (1.29–1.55) | 1.14 (1.04–1.26) | 0.88 (0.80–0.97) |
| | | Men | 3.07 (2.87–3.28) | 1.51 (1.40–1.62) | 0.97 (0.90–1.05) | 1.98 (1.76–2.23) | 1.13 (1.00–1.28) | 0.81 (0.71–0.93) |
| | | Women | 1.94 (1.74–2.17) | 1.15 (1.03–1.30) | 0.94 (0.83–1.06) | 1.78 (1.54–2.05) | 1.18 (1.02–1.37) | 1.11 (0.95–1.30) |
| | ≥70 | Overall | 0.93 (0.88–0.98) | 0.74 (0.71–0.78) | 0.74 (0.70–0.78) | 1.19 (1.12–1.27) | 0.78 (0.73–0.83) | 0.75 (0.70–0.80) |
| | | Men | 0.99 (0.92–1.06) | 0.81 (0.76–0.86) | 0.79 (0.73–0.84) | 1.20 (1.10–1.31) | 0.82 (0.76–0.89) | 0.76 (0.70–0.83) |
| | | Women | 0.85 (0.78–0.92) | 0.66 (0.61–0.72) | 0.69 (0.63–0.74) | 1.20 (1.08–1.33) | 0.74 (0.67–0.81) | 0.74 (0.67–0.82) |

| Outcome | Age | Sex | 1-28 days post-vaccination | | | >28 days post vaccination | | |
|---|---|---|---|---|---|---|---|---|
| | | | Unadjusted[1] | Age sex adjusted[2] | Fully adjusted[3] | Unadjusted[1] | Age sex adjusted[2] | Fully adjusted[3] |
| Ischaemic stroke[4] | <70 | Overall | 2.18 (2.05–2.33) | 1.23 (1.14–1.31) | 0.90 (0.83–0.97) | 1.46 (1.32–1.62) | 1.00 (0.89–1.11) | 0.80 (0.72–0.90) |
| | | Men | 3.05 (2.80–3.32) | 1.38 (1.26–1.51) | 0.95 (0.86–1.05) | 2.40 (2.08–2.77) | 1.26 (1.09–1.46) | 0.91 (0.78–1.07) |
| | | Women | 1.76 (1.59–1.96) | 1.05 (0.94–1.17) | 0.85 (0.76–0.95) | 1.18 (1.01–1.38) | 0.77 (0.66–0.90) | 0.73 (0.61–0.86) |
| | ≥70 | Overall | 0.88 (0.84–0.93) | 0.65 (0.63–0.68) | 0.71 (0.68–0.75) | 1.13 (1.06–1.19) | 0.64 (0.60–0.67) | 0.69 (0.66–0.73) |
| | | Men | 0.94 (0.88–1.00) | 0.71 (0.66–0.75) | 0.77 (0.72–0.82) | 1.17 (1.07–1.27) | 0.69 (0.64–0.75) | 0.75 (0.69–0.81) |
| | | Women | 0.84 (0.78–0.89) | 0.61 (0.58–0.65) | 0.68 (0.63–0.72) | 1.09 (1.01–1.19) | 0.60 (0.56–0.65) | 0.66 (0.61–0.71) |
| Other arterial | <70 | Overall | 1.72 (1.28–2.31) | 0.95 (0.70–1.30) | 0.69 (0.50–0.94) | 1.63 (1.10–2.43) | 1.13 (0.74–1.72) | 0.85 (0.55–1.32) |
| | | Men | 2.31 (1.58–3.37) | 0.99 (0.66–1.49) | 0.71 (0.47–1.07) | 3.22 (2.02–5.13) | 1.56 (0.95–2.58) | 1.16 (0.69–1.96) |
| | | Women | 1.46 (0.90–2.37) | 0.88 (0.54–1.45) | 0.69 (0.42–1.15) | 0.93 (0.43–1.99) | 0.65 (0.30–1.43) | 0.59 (0.26–1.33) |
| | ≥70 | Overall | 0.59 (0.46–0.76) | 0.51 (0.40–0.65) | 0.52 (0.41–0.67) | 0.64 (0.47–0.85) | 0.47 (0.35–0.62) | 0.46 (0.34–0.62) |
| | | Men | 0.49 (0.35–0.69) | 0.46 (0.32–0.65) | 0.47 (0.33–0.66) | 0.52 (0.37–0.74) | 0.46 (0.32–0.66) | 0.44 (0.31–0.64) |
| | | Women | 0.74 (0.51–1.08) | 0.59 (0.41–0.84) | 0.60 (0.42–0.85) | 0.83 (0.50–1.37) | 0.50 (0.31–0.79) | 0.49 (0.30–0.78) |
| **Haematological events** | | | | | | | | |
| Any thrombocytopenia | <70 | Overall | 2.10 (1.60–2.77) | 1.48 (1.11–1.97) | 1.00 (0.75–1.34) | 2.03 (1.42–2.92) | 1.54 (1.07–2.23) | 0.97 (0.66–1.41) |
| | | Men | 3.31 (2.23–4.91) | 2.07 (1.35–3.16) | 1.19 (0.78–1.83) | 2.95 (1.61–5.42) | 2.02 (1.08–3.79) | 0.93 (0.49–1.78) |
| | | Women | 1.50 (1.01–2.22) | 1.17 (0.78–1.74) | 0.97 (0.65–1.46) | 1.58 (1.00–2.50) | 1.32 (0.83–2.08) | 1.14 (0.72–1.80) |
| | ≥70 | Overall | 0.85 (0.64–1.13) | 0.72 (0.54–0.95) | 0.68 (0.51–0.90) | 1.22 (0.87–1.71) | 0.90 (0.64–1.25) | 0.79 (0.56–1.12) |
| | | Men | 0.87 (0.61–1.26) | 0.73 (0.51–1.05) | 0.68 (0.47–0.97) | 1.55 (1.03–2.32) | 1.12 (0.75–1.67) | 0.95 (0.62–1.46) |
| | | Women | 0.82 (0.53–1.29) | 0.71 (0.46–1.09) | 0.68 (0.44–1.07) | 0.84 (0.48–1.48) | 0.63 (0.36–1.09) | 0.57 (0.32–1.02) |
| **Other events** | | | | | | | | |
| Haemorrhagic stroke[5] | <70 | Overall | 1.39 (1.13–1.71) | 0.83 (0.67–1.03) | 0.77 (0.62–0.96) | 1.31 (1.00–1.73) | 0.90 (0.68–1.19) | 0.86 (0.65–1.15) |
| | | Men | 1.65 (1.22–2.23) | 0.86 (0.63–1.18) | 0.77 (0.57–1.05) | 1.43 (0.89–2.29) | 0.86 (0.53–1.40) | 0.77 (0.47–1.25) |
| | | Women | 1.28 (0.95–1.72) | 0.80 (0.59–1.08) | 0.78 (0.58–1.05) | 1.34 (0.95–1.88) | 0.89 (0.62–1.26) | 0.92 (0.65–1.31) |
| | ≥70 | Overall | 0.80 (0.69–0.91) | 0.59 (0.52–0.68) | 0.65 (0.57–0.74) | 1.28 (1.09–1.50) | 0.72 (0.62–0.84) | 0.80 (0.68–0.94) |
| | | Men | 0.82 (0.67–1.01) | 0.59 (0.48–0.71) | 0.64 (0.52–0.78) | 1.45 (1.14–1.83) | 0.78 (0.62–0.98) | 0.86 (0.68–1.09) |
| | | Women | 0.78 (0.65–0.94) | 0.60 (0.50–0.72) | 0.66 (0.55–0.79) | 1.16 (0.93–1.44) | 0.68 (0.56–0.84) | 0.76 (0.61–0.93) |
| Mesenteric thrombosis | <70 | Overall | 1.61 (1.10–2.36) | 0.84 (0.56–1.26) | 0.65 (0.43–0.97) | 1.77 (1.11–2.84) | 1.08 (0.66–1.75) | 0.83 (0.50–1.37) |
| | | Men | 2.91 (1.78–4.75) | 1.29 (0.77–2.17) | 0.97 (0.57–1.64) | 2.21 (0.99–4.94) | 1.15 (0.50–2.62) | 0.82 (0.35–1.91) |
| | | Women | 0.91 (0.49–1.67) | 0.55 (0.29–1.03) | 0.45 (0.23–0.86) | 1.61 (0.90–2.88) | 1.09 (0.60–1.98) | 0.94 (0.50–1.75) |
| | ≥70 | Overall | 0.72 (0.57–0.90) | 0.54 (0.43–0.67) | 0.54 (0.44–0.68) | 1.06 (0.81–1.37) | 0.61 (0.48–0.78) | 0.59 (0.45–0.76) |
| | | Men | 0.57 (0.37–0.86) | 0.41 (0.27–0.61) | 0.42 (0.28–0.62) | 0.82 (0.53–1.29) | 0.44 (0.29–0.66) | 0.42 (0.27–0.65) |
| | | Women | 0.80 (0.61–1.04) | 0.61 (0.47–0.80) | 0.62 (0.48–0.81) | 1.18 (0.86–1.63) | 0.72 (0.53–0.98) | 0.70 (0.51–0.96) |
| Lower limb fracture | <70 | Overall | 1.44 (1.31–1.59) | 1.03 (0.93–1.14) | 0.93 (0.84–1.02) | 1.24 (1.08–1.42) | 0.95 (0.83–1.09) | 0.89 (0.78–1.03) |
| | | Men | 1.19 (0.99–1.42) | 0.94 (0.78–1.12) | 0.85 (0.71–1.02) | 0.99 (0.74–1.31) | 0.83 (0.62–1.11) | 0.80 (0.60–1.07) |
| | | Women | 1.49 (1.33–1.67) | 1.08 (0.96–1.21) | 0.98 (0.87–1.10) | 1.27 (1.09–1.48) | 1.00 (0.85–1.17) | 0.94 (0.80–1.11) |
| | ≥70 | Overall | 0.99 (0.93–1.05) | 0.65 (0.62–0.69) | 0.69 (0.66–0.73) | 1.48 (1.38–1.59) | 0.67 (0.63–0.72) | 0.71 (0.66–0.76) |
| | | Men | 0.95 (0.85–1.06) | 0.58 (0.52–0.64) | 0.63 (0.57–0.70) | 1.60 (1.40–1.83) | 0.65 (0.57–0.73) | 0.69 (0.61–0.79) |
| | | Women | 1.00 (0.93–1.07) | 0.68 (0.64–0.73) | 0.72 (0.67–0.77) | 1.42 (1.30–1.55) | 0.68 (0.63–0.73) | 0.71 (0.65–0.77) |
| Death | <70 | Overall | 0.74 (0.69–0.79) | 0.35 (0.32–0.37) | 0.24 (0.22–0.26) | 0.91 (0.84–1.00) | 0.52 (0.48–0.57) | 0.33 (0.30–0.37) |
| | | Men | 1.06 (0.97–1.17) | 0.39 (0.36–0.43) | 0.28 (0.25–0.31) | 1.62 (1.44–1.82) | 0.69 (0.61–0.79) | 0.44 (0.39–0.51) |
| | | Women | 0.56 (0.50–0.63) | 0.29 (0.26–0.33) | 0.22 (0.19–0.25) | 0.66 (0.58–0.75) | 0.38 (0.34–0.44) | 0.28 (0.25–0.33) |
| | ≥70 | Overall | 0.22 (0.22–0.23) | 0.16 (0.16–0.16) | 0.19 (0.19–0.20) | 0.30 (0.29–0.31) | 0.16 (0.16–0.16) | 0.19 (0.18–0.19) |
| | | Men | 0.23 (0.22–0.24) | 0.16 (0.15–0.16) | 0.19 (0.18–0.20) | 0.33 (0.32–0.34) | 0.16 (0.15–0.17) | 0.19 (0.18–0.19) |
| | | Women | 0.21 (0.20–0.22) | 0.16 (0.16–0.17) | 0.20 (0.19–0.20) | 0.27 (0.26–0.28) | 0.16 (0.15–0.17) | 0.19 (0.18–0.19) |

[1]Stratifed by region

[2]Adjusted for age, age$^2$, sex and stratified by region

[3]Adjusted for age, age$^2$, sex, ethnicity, deprivation, smoking, previous diagnosis of cancer, number of unique diseases in the last year, surgery in the last year, obesity, liver disease; additionally for women aged <70, history of depression and total number of types of medication (by BNF chapters); additionally for men aged <70, history of SARS-CoV2 infection and thrombocytopenia; and additionally for men and women aged ≥70, chronic kidney disease and dementia. Venous events adjusted for: anticoagulant medication, combined oral contraceptive medication, hormone replacement therapy medication, history of PE or DVT, and history of coronavirus infection. Arterial thromboses adjusted for: diabetes, hypertension, smoking, antiplatelet medication, blood pressure lowering medication, lipid lowering medication, anticoagulant medication, history of stroke, and history of MI. All analyses stratified by region.

[4]Ischaemic stroke: including ischaemic stroke, stroke of uncertain cause, and spinal stroke

[5]Hemorrhagic stroke: including intracranial hemorrhage and subarachnoid hemorrhage
