# Supplementary material for: Association of COVID-19 vaccines ChAdOx1 and BNT162b2 with major venous, arterial, or thrombocytopenic events: A population-based cohort study of 46 million adults in England
Source: PLoS Med. 2022 Feb 22;19(2):e1003926. doi: 10.1371/journal.pmed.1003926 (PMC8863280; doi:10.1371/journal.pmed.1003926)
Supplement: S2 Table — (PDF) [file pmed.1003926.s004.pdf]

**S2 Table (a) Fully adjusted hazard ratios (HRs) for the association of ChAdOx1-S and BNT162b2 with venous events 1-28 and >28 days after vaccination, within pre-specified subgroups. P values for between subgroup differences were derived using Wald tests. OCP: oral contraceptive. HRT: hormone replacement therapy**

| | | ChAdOx1-S vaccine | | | | | BNT162b2 vaccine | | | | |
|---|---|---|---|---|---|---|---|---|---|---|---|
| | | N (Events) | Post vaccination ≤28 days | | Post vaccination >28 days | | N (Events) | Post vaccination ≤28 days | | Post vaccination >28 days | |
| **Subgroup** | | **3001** | HR (95% CI)[1] | P value | HR (95% CI)[1] | P value | **3133** | HR (95% CI)[1] | P value | HR (95% CI)[1] | P value |
| **Age groups** | 18-29 | 40 | 2.30 (1.63–3.25) | <0.0001 | 1.29 (0.58–2.90) | 0.003 | 29 | 0.99 (0.60–1.62) | 0.005 | 0.99 (0.57–1.71) | 0.839 |
| | 30-39 | 63 | 1.33 (1.01–1.75) | | 0.85 (0.46–1.59) | | 60 | 0.90 (0.65–1.25) | | 0.71 (0.47–1.07) | |
| | 40-49 | 145 | 1.32 (1.09–1.61) | | 1.52 (1.09–2.13) | | 122 | 0.96 (0.77–1.21) | | 0.75 (0.56–1.01) | |
| | 50-59 | 270 | 0.93 (0.81–1.07) | | 1.00 (0.77–1.30) | | 237 | 0.78 (0.66–0.92) | | 0.69 (0.55–0.86) | |
| | 60-69 | 618 | 0.87 (0.79–0.96) | | 0.89 (0.73–1.07) | | 359 | 0.78 (0.69–0.89) | | 0.67 (0.55–0.83) | |
| | 70-79 | 1124 | 0.75 (0.69–0.82) | | 0.79 (0.71–0.88) | | 823 | 0.69 (0.62–0.77) | | 0.73 (0.65–0.82) | |
| | 80-89 | 546 | 0.79 (0.69–0.89) | | 0.73 (0.63–0.84) | | 1255 | 0.63 (0.56–0.70) | | 0.67 (0.61–0.74) | |
| | >90 | 195 | 0.76 (0.62–0.94) | | 0.75 (0.59–0.95) | | 248 | 0.80 (0.65–0.99) | | 0.67 (0.55–0.81) | |
| **Sex** | Male | 1338 | 0.81 (0.75–0.87) | 0.029 | 0.74 (0.66–0.82) | 0.005 | 1423 | 0.70 (0.64–0.76) | 0.150 | 0.66 (0.60–0.72) | 0.024 |
| | Female | 1663 | 0.90 (0.84–0.97) | | 0.89 (0.81–0.98) | | 1710 | 0.75 (0.70–0.81) | | 0.74 (0.69–0.81) | |
| **Ethnicity** | White | 2856 | 0.86 (0.82–0.91) | 0.018 | 0.82 (0.76–0.89) | 0.429 | 2977 | 0.72 (0.68–0.77) | 0.002 | 0.71 (0.66–0.76) | 0.691 |
| | Asian or Asian British | 38 | 0.51 (0.34–0.77) | | 0.66 (0.38–1.14) | | 56 | 0.69 (0.48–0.98) | | 0.57 (0.38–0.86) | |
| | Black or Black British | 53 | 0.91 (0.66–1.25) | | 0.62 (0.35–1.10) | | 42 | 0.62 (0.41–0.94) | | 0.61 (0.39–0.97) | |
| | Mixed | 10 | 0.47 (0.21–1.06) | | 0.76 (0.28–2.06) | | 12 | 0.74 (0.36–1.51) | | 0.43 (0.16–1.17) | |
| | Other Ethnic Groups | 17 | 0.73 (0.37–1.43) | | 1.63 (0.80–3.32) | | 23 | 1.30 (0.75–2.24) | | 0.94 (0.48–1.84) | |
| | Unknown | 21 | 1.11 (0.65–1.89) | | 1.14 (0.50–2.58) | | 15 | 0.74 (0.36–1.51) | | 0.68 (0.32–1.47) | |
| | Missing | 6 | 2.59 (1.05–6.42) | | 1.29 (0.18–9.32) | | 8 | 3.85 (1.67–8.87) | | 1.33 (0.33–5.43) | |
| **Deprivation** | 1-2 (least) | 564 | 0.76 (0.68–0.85) | 0.138 | 0.76 (0.65–0.89) | 0.43 | 489 | 0.65 (0.57–0.74) | 0.095 | 0.54 (0.47–0.62) | <0.0001 |
| | 3-4 | 560 | 0.86 (0.77–0.96) | | 0.80 (0.68–0.93) | | 576 | 0.76 (0.67–0.85) | | 0.70 (0.62–0.80) | |
| | 5-6 | 625 | 0.90 (0.81–1.00) | | 0.82 (0.71–0.96) | | 606 | 0.70 (0.62–0.79) | | 0.67 (0.59–0.76) | |
| | 7-8 | 632 | 0.87 (0.78–0.97) | | 0.80 (0.69–0.93) | | 687 | 0.73 (0.65–0.82) | | 0.71 (0.63–0.80) | |
| | 9-10 (most) | 609 | 0.91 (0.81–1.01) | | 0.92 (0.79–1.06) | | 770 | 0.81 (0.72–0.91) | | 0.88 (0.79–0.98) | |
| **Prior thrombocytopenia** | No | 3001 | 0.86 (0.82–0.91) | 0.030 | 0.82 (0.76–0.89) | 0.127 | 3132 | 0.73 (0.69–0.78) | 0.014 | 0.71 (0.66–0.75) | 0.372 |
| | Yes | 0 | 0.54 (0.35–0.82) | | 0.54 (0.31–0.93) | | <5 | 0.37 (0.21–0.63) | | 0.58 (0.38–0.89) | |
| **Prior thrombophilia** | No | 2986 | 0.86 (0.81–0.91) | 0.093 | 0.82 (0.76–0.89) | 0.130 | 3116 | 0.73 (0.69–0.77) | 0.254 | 0.70 (0.66–0.75) | 0.924 |
| | Yes | 15 | 0.50 (0.27–0.94) | | 0.38 (0.14–1.03) | | 17 | 0.48 (0.23–0.99) | | 0.68 (0.34–1.37) | |
| **Prior venous event** | No | 3001 | 0.95 (0.90–1.01) | <0.0001 | 0.96 (0.88–1.05) | <0.0001 | 3133 | 0.80 (0.76–0.86) | <0.0001 | 0.78 (0.73–0.84) | <0.0001 |
| | Yes | 0 | 0.54 (0.48–0.61) | | 0.42 (0.35–0.50) | | 0 | 0.44 (0.38–0.51) | | 0.44 (0.38–0.51) | |
| **Prior COVID infection** | No | 2839 | 0.88 (0.83–0.93) | <0.001 | 0.85 (0.79–0.93) | <0.0001 | 3012 | 0.74 (0.70–0.79) | 0.001 | 0.72 (0.68–0.77) | <0.001 |
| | Yes | 162 | 0.60 (0.49–0.74) | | 0.49 (0.37–0.64) | | 121 | 0.47 (0.36–0.61) | | 0.45 (0.34–0.58) | |
| **OCP** | No | 2996 | 1.15 (0.99–1.35) | 0.382 | 1.03 (0.77–1.39) | 0.786 | 3129 | 0.84 (0.70–1.00) | 0.195 | 0.77 (0.61–0.97) | 0.270 |
| | Yes | 5 | 0.69 (0.22–2.17) | | 1.26 (0.31–5.09) | | <5 | 0.33 (0.08–1.34) | | 0.34 (0.08–1.41) | |
| **HRT** | No | 2972 | 0.78 (0.73–0.83) | 0.514 | 0.75 (0.69–0.82) | 0.532 | 3112 | 0.69 (0.65–0.73) | 0.554 | 0.66 (0.62–0.71) | 0.949 |
| | Yes | 29 | 0.92 (0.56–1.50) | | 0.96 (0.45–2.05) | | 21 | 0.57 (0.30–1.08) | | 0.65 (0.34–1.23) | |
| **Anticoagulant** | No | 2759 | 0.98 (0.92–1.03) | <0.0001 | 0.98 (0.90–1.07) | <0.0001 | 2875 | 0.84 (0.79–0.89) | <0.0001 | 0.83 (0.77–0.89) | <0.0001 |
| | Yes | 242 | 0.34 (0.29–0.40) | | 0.28 (0.22–0.35) | | 258 | 0.25 (0.21–0.31) | | 0.27 (0.22–0.32) | |
| **Antiplatelet** | No | 2503 | 0.89 (0.84–0.95) | <0.001 | 0.84 (0.77–0.92) | 0.081 | 2513 | 0.75 (0.71–0.80) | 0.011 | 0.72 (0.67–0.77) | 0.220 |
| | Yes | 498 | 0.69 (0.61–0.78) | | 0.73 (0.62–0.85) | | 620 | 0.62 (0.55–0.71) | | 0.66 (0.58–0.75) | |
| **Prior MI or stroke** | No | 2603 | 0.88 (0.83–0.93) | 0.004 | 0.87 (0.80–0.95) | <0.0001 | 2680 | 0.74 (0.70–0.79) | 0.021 | 0.72 (0.67–0.77) | 0.086 |
| | Yes | 398 | 0.71 (0.62–0.82) | | 0.58 (0.48–0.69) | | 453 | 0.62 (0.53–0.72) | | 0.63 (0.55–0.73) | |
| **Prior diabetes** | No | 2363 | 0.88 (0.83–0.93) | 0.053 | 0.85 (0.78–0.93) | 0.034 | 2456 | 0.74 (0.70–0.79) | 0.149 | 0.71 (0.66–0.76) | 0.639 |
| | Yes | 638 | 0.78 (0.70–0.87) | | 0.72 (0.62–0.83) | | 677 | 0.67 (0.60–0.76) | | 0.69 (0.61–0.78) | |

[1] adjusted

**S2 Table (b) Fully adjusted hazard ratios (HRs) for the association of ChAdOx1-S and BNT162b2 with arterial events 1-28 and >28 days after vaccination, within pre-specified subgroups. P values for between subgroup differences were derived using Wald tests. OCP: oral contraceptive. HRT: hormone replacement therapy**

| | | **ChAdOx1-S vaccine** | | | | | **BNT162b2 vaccine** | | | | |
|---|---|---|---|---|---|---|---|---|---|---|---|
| | | N (Events) | Post vaccination ≤28 days | | Post vaccination >28 days | | N (Events) | Post vaccination ≤28 days | | Post vaccination >28 days | |
| **Subgroup** | **All arterial** | **12890** | HR (95% CI)[1] | P value | HR (95% CI)[1] | P value | **16759** | HR (95% CI)[1] | P value | HR (95% CI)[1] | P value |
| **Age groups** | 18-29 | 12 | 1.72 (0.89–3.33) | | 1.84 (0.59–5.73) | | 21 | 2.01 (1.13–3.57) | | 1.85 (0.96–3.59) | |
| | 30-39 | 54 | 1.51 (1.11–2.06) | | 1.54 (0.87–2.72) | | 67 | 1.36 (1.00–1.86) | | 1.09 (0.74–1.61) | |
| | 40-49 | 277 | 1.37 (1.20–1.57) | | 1.29 (0.99–1.69) | | 284 | 1.17 (1.01–1.36) | | 0.86 (0.70–1.05) | |
| | 50-59 | 963 | 0.98 (0.91–1.05) | <0.0001 | 0.89 (0.76–1.04) | 0.031 | 1062 | 1.06 (0.98–1.14) | <0.0001 | 0.81 (0.72–0.91) | <0.0001 |
| | 60-69 | 2442 | 0.85 (0.81–0.90) | | 0.90 (0.82–0.99) | | 1705 | 0.89 (0.84–0.95) | | 0.83 (0.76–0.92) | |
| | 70-79 | 5030 | 0.90 (0.86–0.94) | | 0.93 (0.88–0.99) | | 3993 | 0.85 (0.81–0.89) | | 0.91 (0.86–0.96) | |
| | 80-89 | 2826 | 0.86 (0.81–0.91) | | 0.88 (0.82–0.94) | | 7733 | 0.74 (0.71–0.78) | | 0.79 (0.76–0.82) | |
| | >90 | 1286 | 0.85 (0.78–0.93) | | 0.86 (0.78–0.94) | | 1894 | 0.72 (0.66–0.79) | | 0.78 (0.73–0.84) | |
| **Sex** | Male | 7116 | 0.78 (0.75–0.80) | <0.0001 | 0.75 (0.71–0.79) | <0.0001 | 9292 | 0.77 (0.74–0.79) | <0.0001 | 0.71 (0.68–0.73) | <0.0001 |
| | Female | 5774 | 0.89 (0.86–0.93) | | 0.87 (0.83–0.92) | | 7467 | 0.85 (0.82–0.88) | | 0.89 (0.85–0.92) | |
| **Ethnicity** | White | 11729 | 0.82 (0.79–0.84) | | 0.80 (0.77–0.84) | | 15492 | 0.80 (0.78–0.82) | | 0.79 (0.76–0.81) | |
| | Asian or Asian British | 647 | 0.84 (0.76–0.93) | | 0.74 (0.64–0.86) | | 728 | 0.75 (0.67–0.84) | | 0.63 (0.56–0.71) | |
| | Black or Black British | 217 | 0.90 (0.75–1.07) | | 0.89 (0.70–1.15) | | 190 | 0.77 (0.62–0.94) | | 0.79 (0.64–0.99) | |
| | Mixed | 72 | 1.19 (0.90–1.57) | <0.0001 | 0.56 (0.32–0.98) | 0.180 | 85 | 1.10 (0.82–1.49) | <0.0001 | 0.81 (0.57–1.17) | <0.0001 |
| | Other Ethnic Groups | 113 | 0.93 (0.73–1.18) | | 0.90 (0.64–1.28) | | 121 | 0.89 (0.69–1.15) | | 0.76 (0.57–1.01) | |
| | Unknown | 80 | 0.88 (0.67–1.16) | | 0.75 (0.49–1.16) | | 103 | 0.87 (0.64–1.18) | | 1.09 (0.83–1.44) | |
| | Missing | 32 | 3.01 (1.98–4.57) | | 1.91 (0.89–4.08) | | 40 | 2.87 (1.81–4.56) | | 2.87 (1.80–4.58) | |
| **Deprivation** | 1-2 (least) | 2498 | 0.76 (0.71–0.80) | | 0.71 (0.66–0.77) | <0.0001 | 2788 | 0.75 (0.71–0.79) | | 0.69 (0.65–0.73) | |
| | 3-4 | 2514 | 0.81 (0.76–0.85) | | 0.76 (0.71–0.82) | | 2980 | 0.76 (0.72–0.81) | | 0.74 (0.69–0.78) | |
| | 5-6 | 2683 | 0.85 (0.81–0.90) | <0.001 | 0.79 (0.74–0.85) | | 3489 | 0.78 (0.74–0.83) | <0.0001 | 0.79 (0.75–0.84) | <0.0001 |
| | 7-8 | 2616 | 0.83 (0.78–0.87) | | 0.86 (0.80–0.92) | | 3693 | 0.82 (0.78–0.87) | | 0.83 (0.79–0.88) | |
| | 9-10 (most) | 2541 | 0.89 (0.85–0.95) | | 0.88 (0.82–0.95) | | 3772 | 0.90 (0.85–0.95) | | 0.85 (0.80–0.89) | |
| **Prior thrombocytopenia** | No | 12889 | 0.82 (0.80–0.85) | 0.334 | 0.80 (0.77–0.84) | 0.499 | 16758 | 0.80 (0.78–0.82) | 0.920 | 0.78 (0.76–0.81) | 0.136 |
| | Yes | <5 | 0.75 (0.61–0.91) | | 0.73 (0.57–0.95) | | <5 | 0.81 (0.66–1.00) | | 0.67 (0.54–0.82) | |
| **Prior thrombophilia** | No | 12864 | 0.82 (0.80–0.85) | 0.059 | 0.80 (0.77–0.83) | 0.620 | 16735 | 0.80 (0.78–0.82) | 0.499 | 0.78 (0.76–0.81) | 0.098 |
| | Yes | 26 | 0.48 (0.28–0.84) | | 0.66 (0.32–1.39) | | 24 | 0.66 (0.38–1.15) | | 0.43 (0.21–0.88) | |
| **Prior venous event** | No | 12874 | 0.83 (0.81–0.85) | 0.029 | 0.80 (0.77–0.84) | 0.504 | 16751 | 0.81 (0.78–0.83) | 0.022 | 0.79 (0.76–0.81) | 0.002 |
| | Yes | 16 | 0.73 (0.66–0.82) | | 0.77 (0.67–0.88) | | 8 | 0.70 (0.63–0.79) | | 0.66 (0.59–0.74) | |
| **Prior COVID infection** | No | 12227 | 0.84 (0.81–0.86) | <0.0001 | 0.83 (0.79–0.86) | <0.0001 | 16234 | 0.81 (0.79–0.84) | <0.0001 | 0.80 (0.77–0.82) | <0.0001 |
| | Yes | 663 | 0.63 (0.57–0.70) | | 0.51 (0.45–0.59) | | 525 | 0.57 (0.50–0.65) | | 0.51 (0.45–0.59) | |
| **OCP** | No | 12889 | 0.91 (0.79–1.05) | 0.983 | 0.89 (0.67–1.18) | <0.0001 | 16759 | 0.91 (0.79–1.06) | <0.0001 | 0.94 (0.75–1.16) | <0.0001 |
| | Yes | <5 | 0.89 (0.12–6.51) | | 0.00 (0.00–0.00) | | 0 | 0.00 (0.00–0.00) | | 0.00 (0.00–0.00) | |
| **HRT** | No | 12832 | 0.85 (0.82–0.87) | 0.293 | 0.84 (0.80–0.88) | 0.820 | 16671 | 0.81 (0.78–0.83) | 0.061 | 0.79 (0.76–0.81) | 0.070 |
| | Yes | 58 | 1.01 (0.73–1.40) | | 0.79 (0.45–1.37) | | 88 | 1.10 (0.79–1.51) | | 1.08 (0.77–1.52) | |
| **Anticoagulant** | No | 10726 | 0.84 (0.82–0.87) | <0.001 | 0.82 (0.78–0.85) | 0.015 | 13715 | 0.83 (0.81–0.86) | <0.0001 | 0.80 (0.77–0.83) | <0.001 |
| | Yes | 2164 | 0.74 (0.69–0.79) | | 0.74 (0.68–0.80) | | 3044 | 0.66 (0.62–0.70) | | 0.71 (0.67–0.76) | |
| **Antiplatelet** | No | 7636 | 0.99 (0.96–1.03) | <0.0001 | 1.06 (1.01–1.11) | <0.0001 | 9770 | 0.96 (0.93–0.99) | <0.0001 | 1.05 (1.01–1.09) | <0.0001 |
| | Yes | 5254 | 0.65 (0.63–0.68) | | 0.59 (0.55–0.62) | | 6989 | 0.65 (0.63–0.67) | | 0.55 (0.53–0.57) | |
| **Prior MI or stroke** | No | 6931 | 1.09 (1.05–1.13) | <0.0001 | 1.18 (1.12–1.24) | <0.0001 | 9428 | 1.08 (1.04–1.12) | <0.0001 | 1.22 (1.17–1.26) | <0.0001 |
| | Yes | 5959 | 0.64 (0.61–0.66) | | 0.60 (0.57–0.63) | | 7331 | 0.60 (0.57–0.62) | | 0.51 (0.49–0.53) | |
| **Prior diabetes** | No | 8742 | 0.85 (0.83–0.88) | <0.0001 | 0.84 (0.80–0.88) | <0.001 | 11689 | 0.84 (0.82–0.87) | <0.0001 | 0.82 (0.79–0.85) | <0.0001 |
| | Yes | 4148 | 0.77 (0.73–0.80) | | 0.73 (0.69–0.78) | | 5070 | 0.72 (0.69–0.76) | | 0.70 (0.67–0.74) | |

[1] adjusted
