# Supplementary material for: Association of COVID-19 vaccines ChAdOx1 and BNT162b2 with major venous, arterial, or thrombocytopenic events: A population-based cohort study of 46 million adults in England
Source: PLoS Med. 2022 Feb 22;19(2):e1003926. doi: 10.1371/journal.pmed.1003926 (PMC8863280; doi:10.1371/journal.pmed.1003926)
Supplement: S3 Table — Only percentage are presented, as disclosure control does not allow numbers <5 to be released. ICVT, intracranial venous thrombosis. (PDF) [file pmed.1003926.s005.pdf]

S3 table. Characteristics of patient who had an ICVT event before and after vaccination. Only percentage are presented, as disclosure control does not allow numbers <5 to be released

		ICVT before vaccination %	ICVT after ChAdOx1, %	ICVT after BNT162b, %	p-value[1]
N		<210	<40	<20	<0.0001
Sex	Male	40	33	21	0.24
	Female	60	67	79	
Age	18 - 29	19	17	0	<0.0001
	30 - 49	42	22	26	
	50 - 69	30	36	16	
	70 - 79	6	11	21	
	80+	3	14	37	
Ethnicity	Asian or Asian British	15	6	0	0.38
	Black or Black British	5	3	0	
	Mixed	2	0	0	
	Other ethnic groups	1	0	0	
	White	75	92	100	
	Unknown or missing	1	0	0	
Deprivation[2]	1 - 2	29	17	11	0.19
	3 - 4	20	14	26	
	5 - 6	25	25	21	
	7 - 8	13	25	16	
	9 - 10	13	19	26	
Smoking status	Current	19	28	11	0.5
	Former	21	17	32	
	Never	57	56	58	
Medical history	Stroke	5	6	11	<0.0001
	MI	3	6	0	0.01
	DVT or PE	8	6	5	<0.0001
	Thrombophilia	1	0	0	0.05
	Coronavirus infection[3]	6	6	0	<0.0001
	Diabetes	10	17	37	<0.0001
	Depression	26	36	16	<0.0001
	Obesity	37	31	42	<0.0001
	Cancer	17	28	37	<0.0001
	COPD	2	11	16	0.78
	Liver disease	<1	3	0	0.61
	CKD	5	11	32	0.25
	Major surgery[4]	18	8	11	<0.0001
	Dementia	2	3	0	0.03
Medications	Antiplatelet	5	6	16	0.01
	BP lowering	15	28	42	<0.0001
	Lipid lowering	12	22	26	<0.0001
	Anticoagulant	10	11	11	<0.0001
	Oral contraceptive	4	0	0	<0.0001
	HRT	1	0	0	0.05
Number of diagnoses[5]	0	71	50	58	0.04
	1 - 5	29	50	42	
Number of medications	0	31	11	2	0.02
	1 - 5	62	83	5	
	6+	7	6	2	

[1] Approximation if Chi squared test included one or more zero counts; [2] Index of Multiple Deprivation deciles where 1 indicates least deprived and 10 indicates most deprived; [3] After 31/12/2019 and prior to 08/12/2020; [4] In the last year; [5] The category '6+' contained no individuals and so was excluded.
