# Supplementary figures and images for: Association of COVID-19 vaccines ChAdOx1 and BNT162b2 with major venous, arterial, or thrombocytopenic events: A population-based cohort study of 46 million adults in England
Source: PLoS Med. 2022 Feb 22;19(2):e1003926. doi: 10.1371/journal.pmed.1003926 (PMC8863280; doi:10.1371/journal.pmed.1003926)

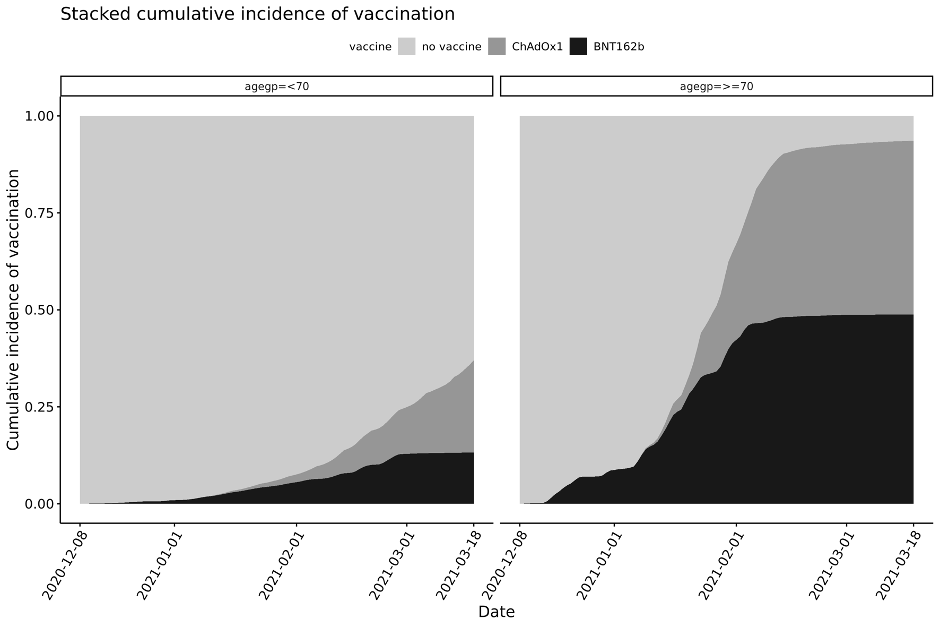

Supplement: S1 Fig — (PNG) [file pmed.1003926.s006.png]

## A Venous

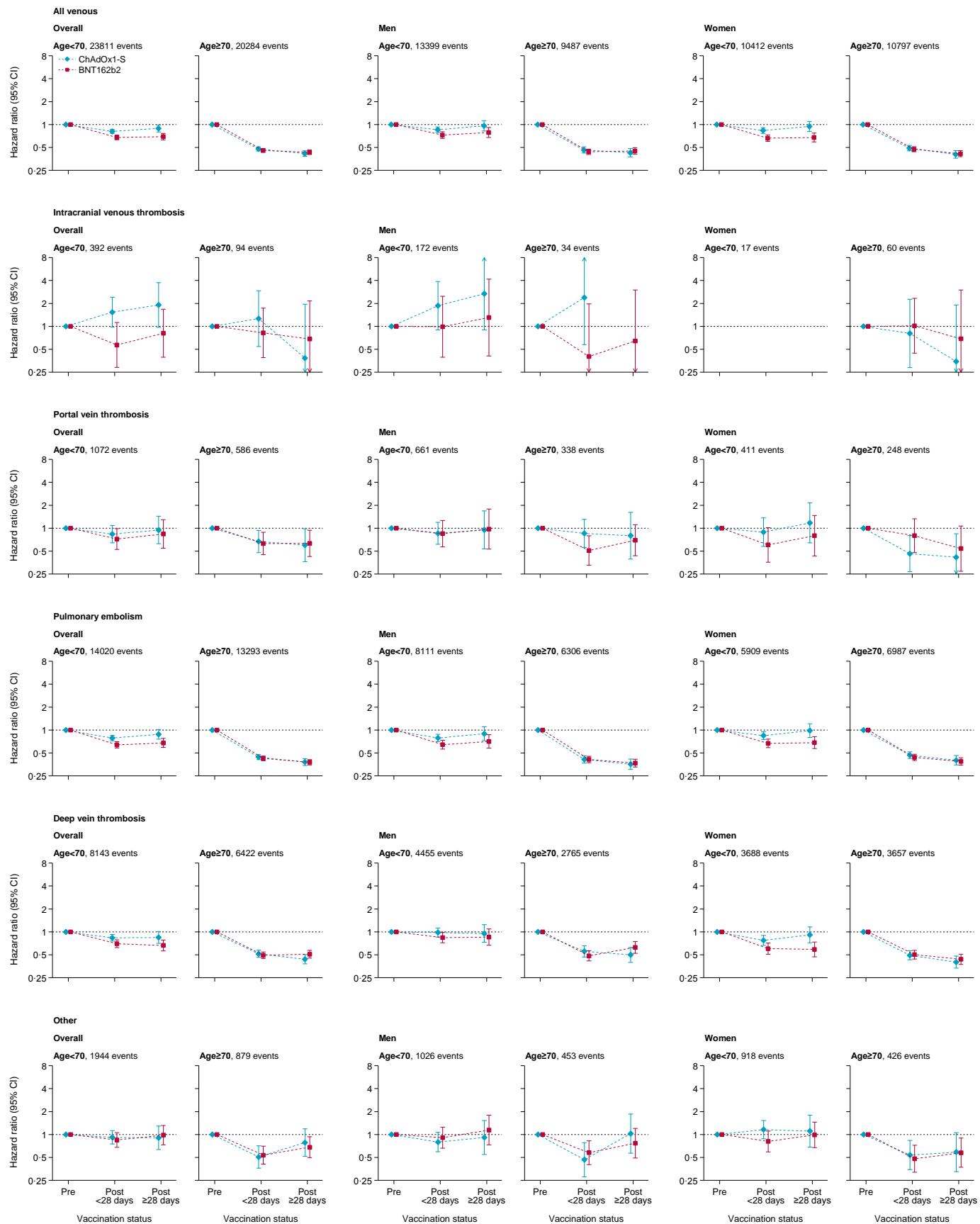

## B Arterial

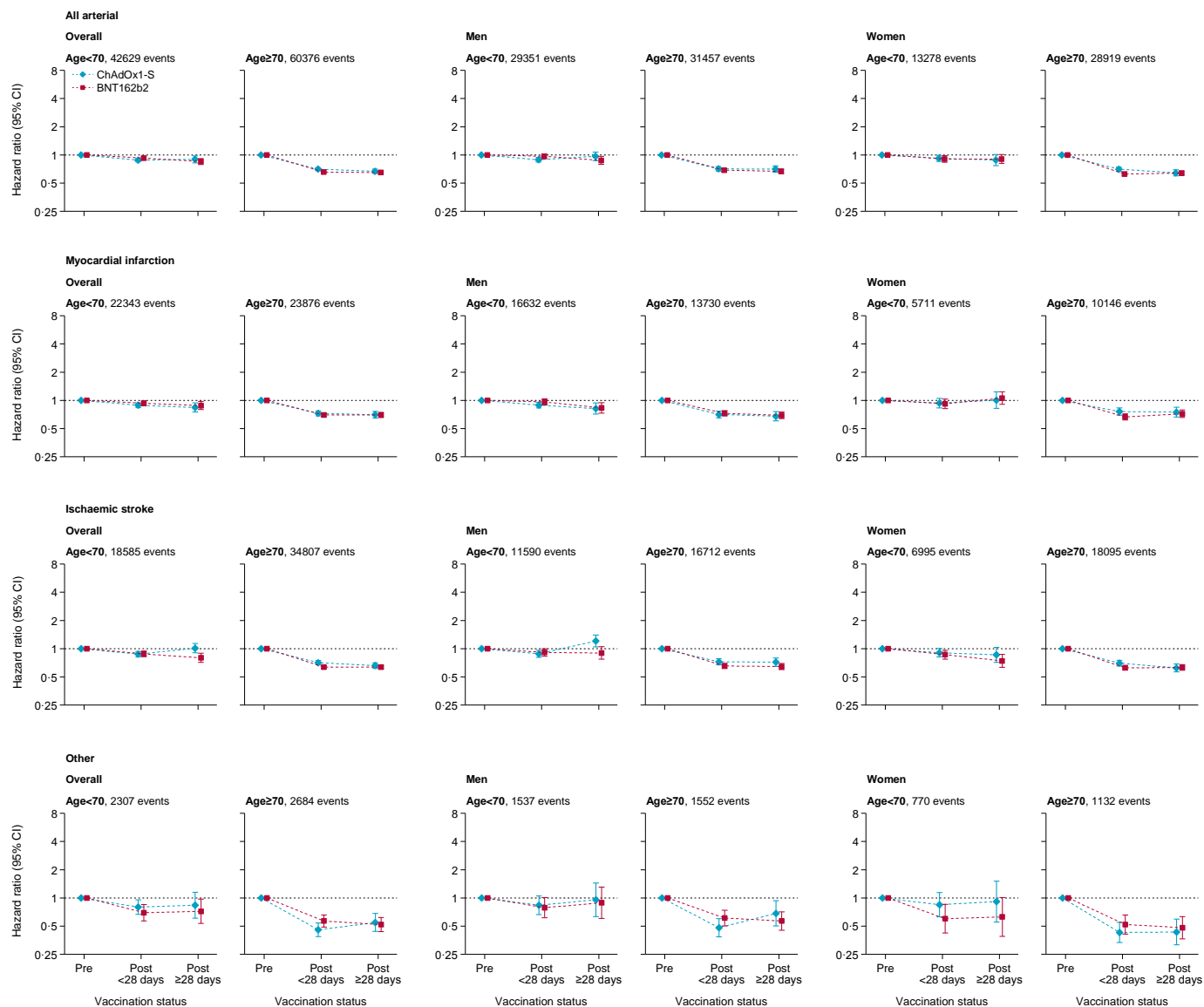

## C Other

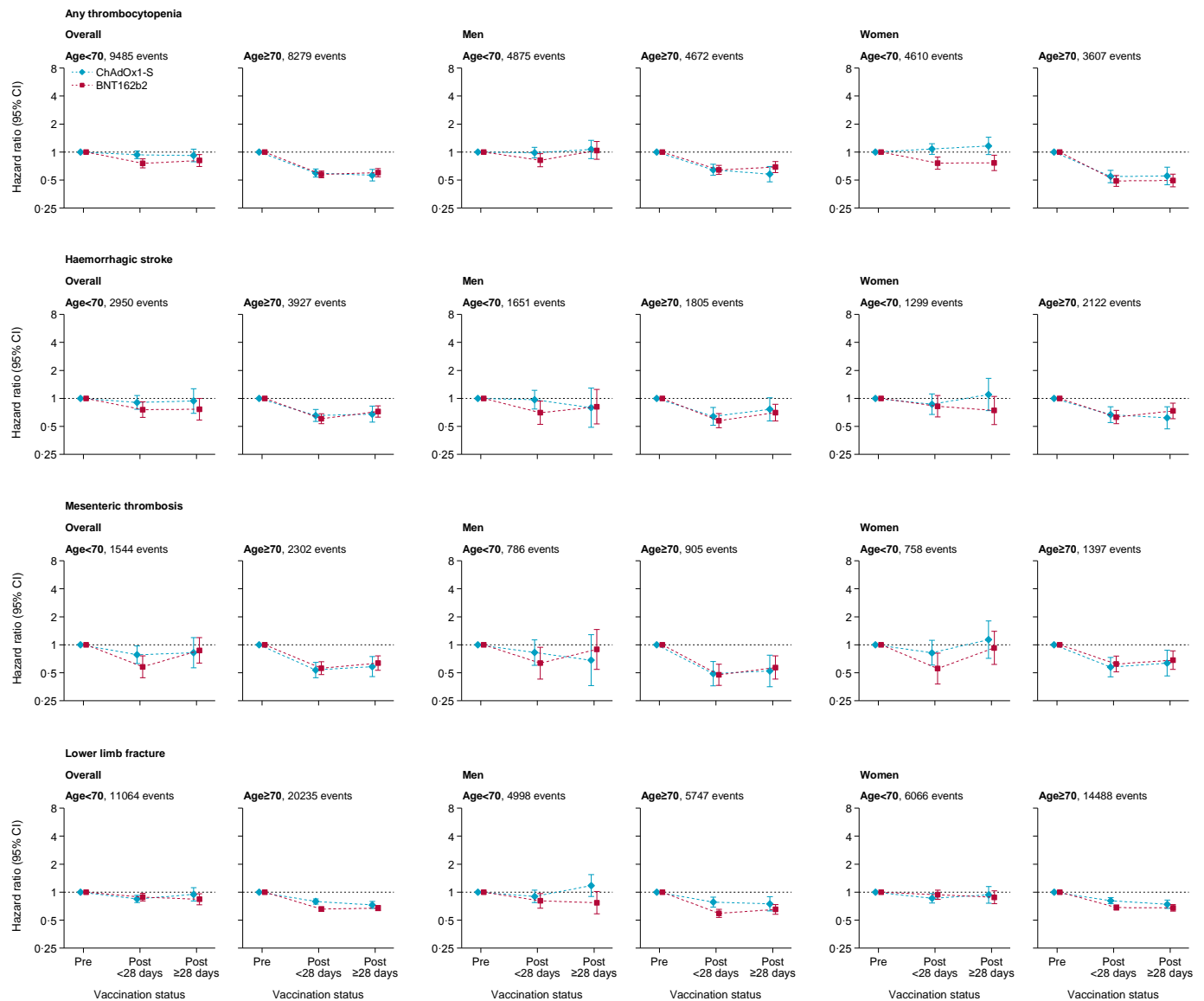

Supplement: S3 Fig — HRs for major (A) arterial and (B) venous thrombotic events and (C) haematological events, other events and lower limb fractures recorded in any position in EHR. CI, confidence interval; EHR, electronic health records; HR, hazard ratio. (PDF) [file pmed.1003926.s008.pdf]

## A Venous

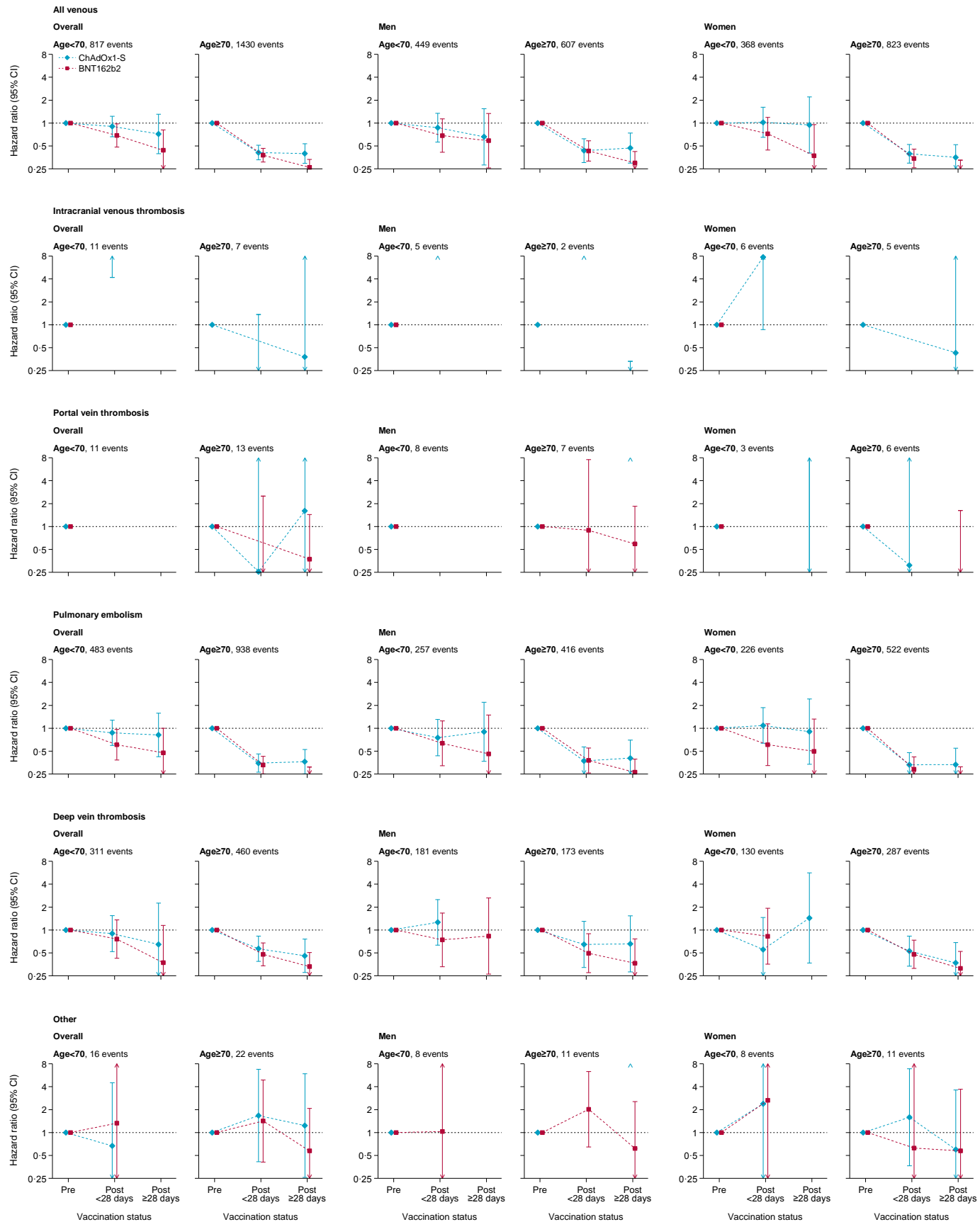

## B Arterial

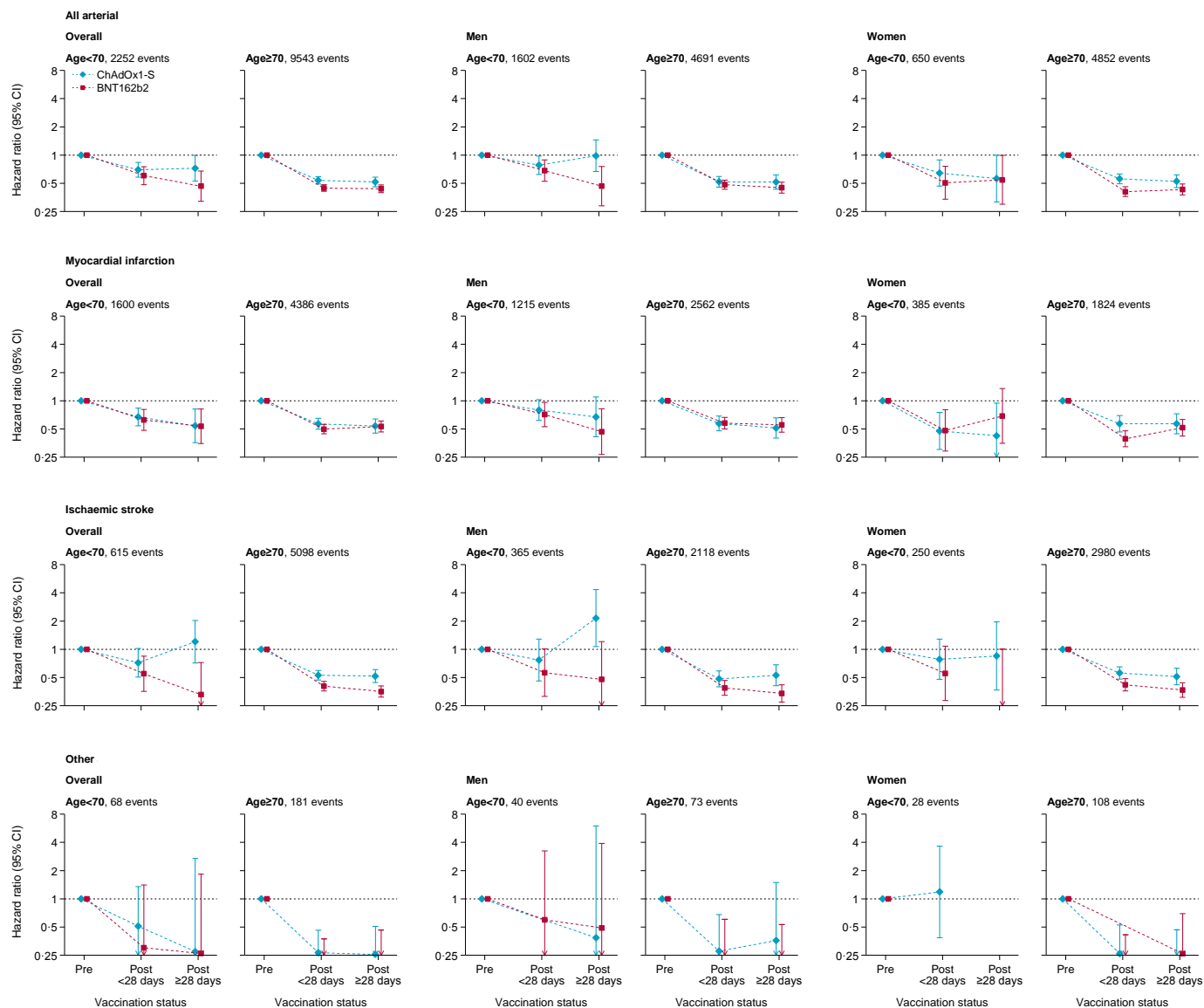

## C Other

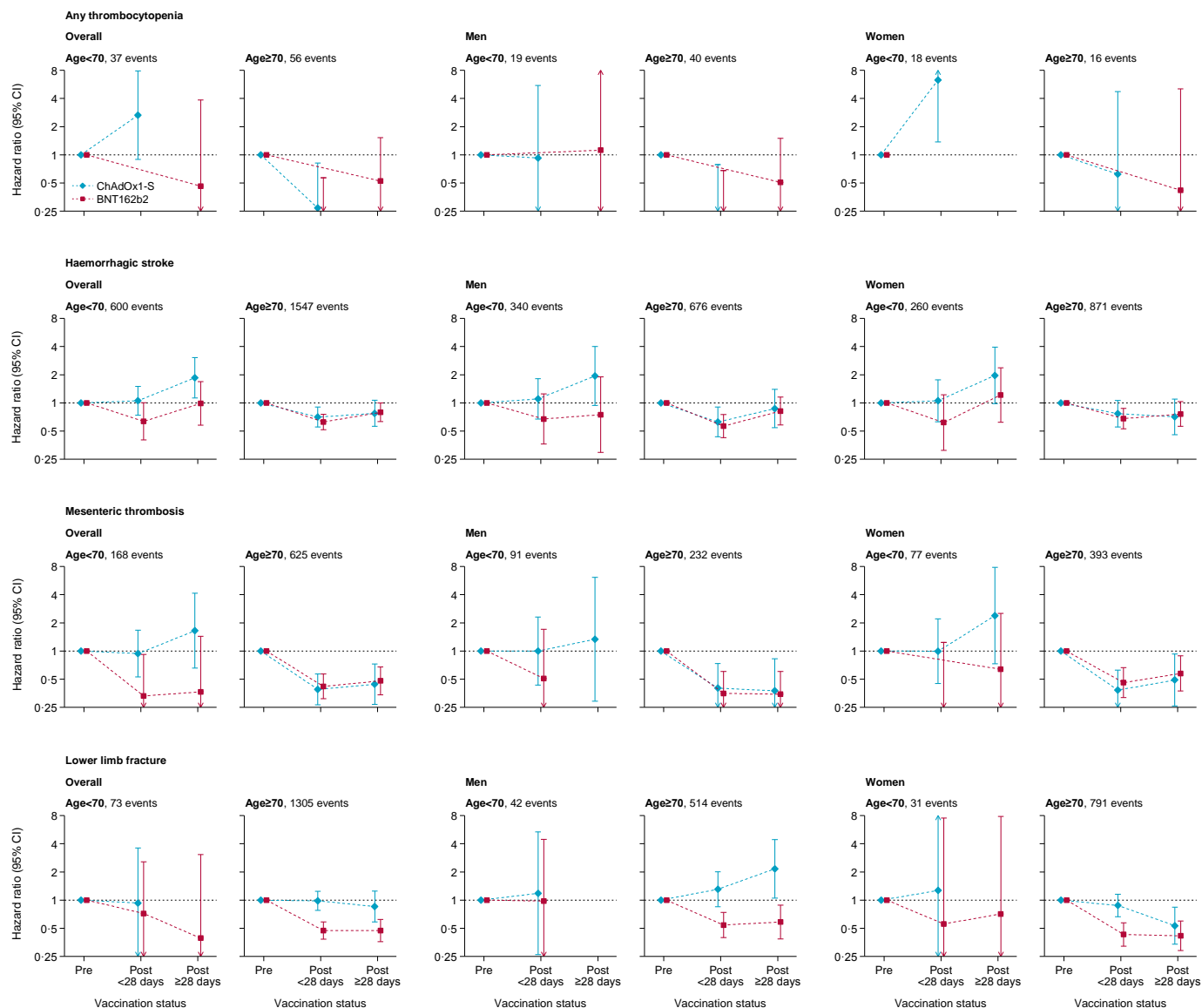

Supplement: S4 Fig — HRs for major (A) arterial and (B) venous thrombotic events and (C) haematological events, other events and fractures recorded in death record or in hospital record in first position followed by death <28 days. CI, confidence interval; HR, hazard ratio. (PDF) [file pmed.1003926.s009.pdf]
