# Supplementary material for: Association of COVID-19 vaccines ChAdOx1 and BNT162b2 with major venous, arterial, or thrombocytopenic events: A population-based cohort study of 46 million adults in England
Source: PLoS Med. 2022 Feb 22;19(2):e1003926. doi: 10.1371/journal.pmed.1003926 (PMC8863280; doi:10.1371/journal.pmed.1003926)
Supplement: S2 Fig — aHR, adjusted HR; CI, confidence interval. (PDF) [file pmed.1003926.s007.pdf]

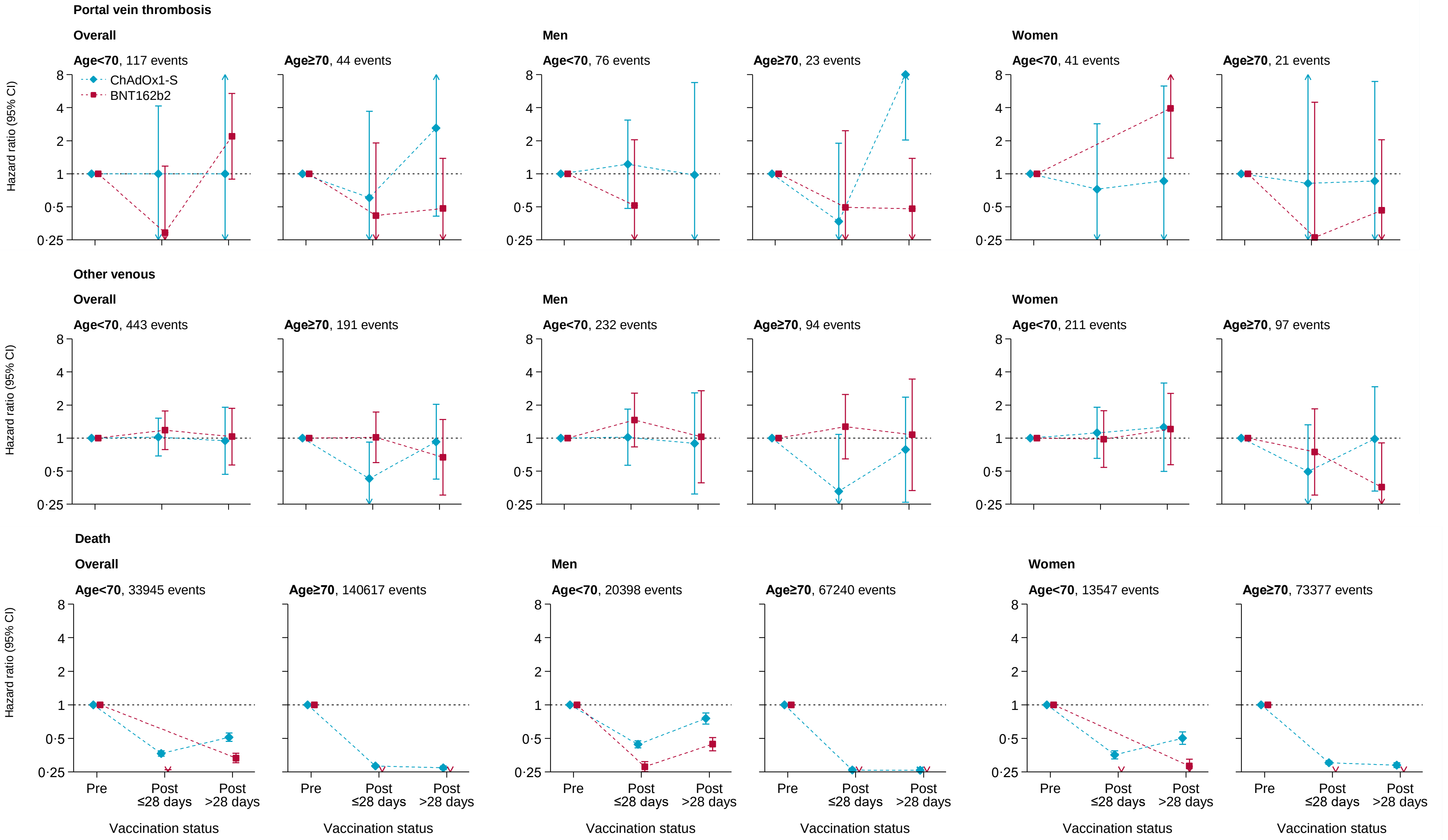

S2 Figure: Adjusted hazard ratios for portal vein thrombosis, other venous events, and death after ChAdOx1-S or BNT162b2 vaccine
