# Supplementary material for: Association of COVID-19 vaccines ChAdOx1 and BNT162b2 with major venous, arterial, or thrombocytopenic events: A population-based cohort study of 46 million adults in England
Source: PLoS Med. 2022 Feb 22;19(2):e1003926. doi: 10.1371/journal.pmed.1003926 (PMC8863280; doi:10.1371/journal.pmed.1003926)
Supplement: Attachment — Submitted filename: Response to reviewers v0.2.docx [file pmed.1003926.s010.docx]

Response to reviewers and editors

**Requests from the editors:**

1. **Throughout the paper, please adapt reference call-outs to the following style: "... intracerebral haemorrhage [1,3,4]." (noting the absence of spaces within the square brackets).**

RESPONSE: Done

2. **Title: Please revise your title according to PLOS Medicine's style. We suggest “Association of COVID-19 vaccines ChAdOx1 and BNT162b2 with major venous, arterial, or thrombocytopenic events: A population-based cohort study of 46 million adults in England” or similar**

RESPONSE:

Done. The title now reads “*Association of COVID-19 vaccines ChAdOx1 and BNT162b2 with major venous, arterial, or thrombocytopenic events: A population-based cohort study of 46 million adults in England”*

3. **Abstract:
   Please combine the Methods and Findings sections into one section, “Methods and Findings”.**

RESPONSE:

Done

4. **Abstract Background: Provide expand on the context of why your study is important. The final sentence should clearly state the study question.**

RESPONSE:

the abstract background now reads:

*“Thromboses in unusual locations after the COVID-19 vaccine ChAdOx1-S have been reported, although there frequency with vaccines of different types is uncertain at a population level. The aim of this study was to estimate the population-level risks of hospitalised thrombocytopenia and major arterial and venous thromboses after COVID-19 vaccination.”*

5. **Abstract Methods and Findings:
   Please provide brief demographic details of the study population (e.g. sex, age, ethnicity, etc)**

RESPONSE:

the first sentence now reads:

*“Of 46 million adults, 23 million (51%) were women; 39 million (84%) were aged <70 years, 3.7 million (8.1%) were Asian or Asian British, 1.6 million (3.5%) were Black or Black British, 36 million (79%) were White, 0.7 million (1.5%) had mixed ethnicity and 1.5 million (3.2%) were of other ethnicity.”*

6. **Please specify the study design i.e. population-based cohort study**

RESPONSE:

First sentence of methods and findings now reads:

*“In this whole-population cohort study, we analysed linked electronic health records from adults living in England”*

7. **Please clarify what is meant by ‘during follow up’**

RESPONSE.

Now reads:

*“21 million (46%) adults had their first vaccination between December 8^th^ 2020 and March 18^th^ 2021.”*

8. **Please provide the incidence rates and/or absolute risks of relevant outcomes, not just aHRs**

RESPONSE:

We have included a paragraph on the absolute incidence rates of arterial and venous events. This does add length to the abstract; if the editors would want to leave these out (as one can see they are substantially confounded, our original reason for not including them), please let us know.

“*The crude incidence rates (per 100,000 person-years) of all venous events were: pre vaccination, 141 (95%CI: 139–143); ≤28 days post ChAdOx1-S, 309 (296–323); >28 days post ChAdOx1-S, 383 (360–407), ≤28 days post BNT162b2-S, 250 (238–263); >28 days post BNT162b2-S 287 (273–302). The crude incidence rates (per 100,000 person-years) of all arterial events were: pre vaccination, 549 (95%CI: 545–554); ≤28 days post ChAdOx1-S, 1274 (1247–1302); >28 days post ChAdOx1-S, 1787 (1736–1839), ≤28 days post BNT162b2-S, 1290 (1262–1318); >28 days post BNT162b2-S, 1595 (1562–1629)*.”

9. **Please include more detail on the important dependent variables that are adjusted for in the analyses.**

RESPONSE:

added the following sentence to the abstract

“*Analyses were performed separately for ages <70 and ≥70 years, and adjusted for age, age^2^, sex, ethnicity ,deprivation and in analyses of venous thromboses: anticoagulant medication, combined oral contraceptive medication, hormone replacement therapy medication, history of PE or DVT, and history of coronavirus infection; and in analyses of arterial thromboses: diabetes, hypertension, smoking, antiplatelet medication, blood pressure lowering medication, lipid lowering medication, anticoagulant medication, history of stroke, and history of MI. Further covariates were selected using backward selection*.”

10. **Lines 74, 76 - please clarify whether these are HRs or aHRs**

RESPONSE

These were adjusted HRs. Now clarified.

11. **In the last sentence of the Abstract Methods and Findings section, please describe 2-3 of  the main limitations of the study's methodology.**

RESPONSE: now added:

*“The main limitations of the study are: (i) that it relies on the accuracy of coded healthcare data to identify exposures, covariates and outcomes; (ii) the use of primary reason for hospital admission to measure outcome which improves the positive predictive value but may lead to an underestimation of incidence; and (iii) potential unmeasured confounding.”*

12. **Abstract Conclusions:
    Please begin your Abstract Conclusions with "In this study, we observed ..." or similar, to summarize the main findings from your study, without overstating your conclusions. Please emphasize what is new and address the implications of your study, being careful to avoid assertions of primacy.**

RESPONSE: now reads:

*“In this study, we observed increases in rates of ICVT and thrombocytopenia after ChAdOx1-S vaccination in adults aged <70 years that were small compared with its effect in reducing COVID-19 morbidity and mortality, although more precise estimates for adults aged <40 years are needed. For people aged ≥70 years, rates of arterial or venous thrombotic, events were generally lower after either vaccine**, suggesting either vaccine is suitable in this age group.”*

13. Author Summary:
    **At this stage, we ask that you include a short, non-technical Author Summary of your research to make findings accessible to a wide audience that includes both scientists and non-scientists. The Author Summary should immediately follow the Abstract in your revised manuscript. This text is subject to editorial change and should be distinct from the scientific abstract. Please see our author guidelines for more information:** [**https://journals.plos.org/plosmedicine/s/revising-your-manuscript#loc-author-summary**](https://journals.plos.org/plosmedicine/s/revising-your-manuscript#loc-author-summary) **In the final bullet point of ‘What Do These Findings Mean?’, please describe the main limitations of the study in non-technical  language.**

RESPONSE:

We have added an author summary to p5

14. **Introduction:
    Please indicate whether your study is novel, being careful to temper assertions of primacy.**

RESPONSE

The cohort study is new, and we have indicated this in the introduction and new data source in the methods

15. Methods:
    **Please state that your study had a prospective protocol / analysis plan early in the Methods section; please include the relevant prospectively written document with your revised manuscript as a Supporting Information file to be published alongside your study. A legend for this file should be included at the end of your manuscript. Changes in the analysis-- including those made in response to peer review comments-- should be identified as such in the Methods section of the paper, with rationale. If a reported analysis was performed based on an interesting but unanticipated pattern in the data, please be clear that the analysis was data-driven.**

RESPONSE:

We now report that the protocol and analysis plan were published in advance, at the start of the methods section, and include the document as a supplementary file. We have indicated deviations from the protocol within the methods section.

16. **Thank you for providing a RECORD checklist. Please add the following statement, or similar, to the Methods: "This study is reported as per the Strengthening the REporting of studies Conducted using Observational Routinely-collected Data (RECORD) guideline (S1 Checklist)." When completing the checklist, please use section and paragraph numbers, rather than page numbers which will likely no longer correspond to the appropriate sections after copy-editing.**
    RESPONSE:

Done

17. **Line 159 - please refer to a specific file in your supporting information e.g. S1 Table**

RESPONSE:

These are listed in the protocol, now this is referenced in the methods.

18. **Results:
    Please define the length of follow up (eg, in mean, SD, and range).**

RESPONSE:

The median follow up was 101 days (the whole follow-up period)

19. **Line 265 - please clarify which vaccination ‘post-vaccinated’ refers to**

RESPONSE:

Now clarified to *“For example, in <70 year-olds the HRs 1-28 days after* ***vaccination with ChAdOx1-S*** *versus unvaccinated were attenuated from 2.81 to 2.27 for ICVT; from 4.85 to 1.71 for thrombocytopenia; and from 3.21 to 0.90 for ischaemic stroke (supplementary table 1).*

20. **Supplementary Table 1 is central to the understanding of the paper. Please incorporate it into the main paper.**

RESPONSE:

Done

21. **Where aHRs are reported, please specify the comparison group.**

RESPONSE:

Done

22. **Discussion:
    Please present and organize the Discussion as follows: a short, clear summary of the article's findings; what the study adds to existing research and where and why the results may differ from previous research; strengths and limitations of the study; implications and next steps for research, clinical practice, and/or public policy; one-paragraph conclusion.**

RESPONSE:

This is the case for most of our discussion. Added one paragraph of conclusion

23. **Line 354 - please temper assertions of primacy by adding ‘to the best of our knowledge’ or similar**

RESPONSE:
Sentence now deleted

24. **Figures:
    Please provide Figures 1 - 3 in your revised manuscript.**

RESPONSE:

Done

25. **Please indicate in the figure caption the meaning of the error bars in Supplementary Figures 2-4**

RESPONSE:

Done

26. **Supplementary Fig. 1 - Please consider avoiding the use of red and green in order to make your figure more accessible to those with colour blindness.**

RESPONSE:

Done – changed to grey scale.

27. **Tables:
    Please define all abbreviations used in the table legend of each table.**

RESPONSE
Done for table 1

28. **Table 1 - please clarify whether this is risk per 100,000 people pre- or post-vaccination**

RESPONSE:
This is risk for the population during follow up, whether or not vaccinated. Clarified with the legend:

*“Numbers of patients analysed and (in parentheses) risk per 100,000* ***people for the whole population during follow up*** *of venous and arterial events and thrombocytopenia (TCP), overall and according to age.”*

29. **Please specify the variables adjusted for in the table legend of Supplementary Table 1.**

RESPONSE:

Now footnote expanded to:

*^3^Adjusted for age, age^2^, sex, ethnicity, deprivation, smoking, previous diagnosis of cancer, number of unique diseases in the last year, surgery in the last year, obesity, liver disease; additionally for women aged <70, history of depression and total number of types of medication (by BNF chapters); additionally for men aged <70, history of SARS-CoV2 infection and thrombocytopenia; and additionally for men and women aged ≥70, chronic kidney disease and dementia. Venous events adjusted for: anticoagulant medication, combined oral contraceptive medication, hormone replacement therapy medication, history of PE or DVT, and history of coronavirus infection. Arterial thromboses adjusted for: diabetes, hypertension, smoking, antiplatelet medication, blood pressure lowering medication, lipid lowering medication, anticoagulant medication, history of stroke, and history of MI. All analyses stratified by region.*

30. **When a p value is given, please specify the statistical test used to determine it in the table legend.**

RESPONSE:

This is done already for the supplementary tables with p values

31. **References:
    Please ensure that journal name abbreviations match those found in the National Center for Biotechnology Information (NCBI) databases, and are appropriately formatted and capitalised.
    Please also see** [**https://journals.plos.org/plosmedicine/s/submission-guidelines#loc-references**](https://journals.plos.org/plosmedicine/s/submission-guidelines#loc-references) **for further details on reference formatting.
    Where websites are cited, please provide date of access.**

RESPONSE:

Done

Supplementary files:
Please see <https://journals.plos.org/plosmedicine/s/supporting-information> for our supporting information guidelines.


**REQUESTS FROM REVIEWERS**

Reviewer #1: I confine my remarks to statistical aspects of this paper. The general approach is fine and appropriate but I have a couple issues to resolve before I can recommend publication.

32. **One is that the authors have the entire population. This makes use of p values and confidence intervals problematic. These statistics are about inference from a sample to a population, but you have the population. My preference would be to remove p values and CIs. Some statisticians disagree and say that you can posit a sort of "super population".  I don't find their arguments persuasive, but I won't reject an article that uses this idea, but it has to be mentioned.**

RESPONSE:

First, we believe that our findings are generalizable to healthcare systems outside England, so that our data should be considered as a (systematic) sample from a wider population of interest. Second, it would be very unhelpful to omit confidence intervals, because these convey the greater imprecision in estimated hazard ratios for rare outcome than those for common outcomes. Third, in our estimation of the association measures, we used a 10% random sample of the individuals without the outcome of interest which will (minimally) affect the precision of the estimates. For these reasons, our strong preference is to retain the confidence intervals in Figures 1-3.

Please note that we have not provided confidence intervals for estimates of the excess risk of IVCT post vaccination, as these statistics are specific to the population of England.

33. **I would like more explanation of why follow-up was split up the way it was.  Usually, categorizing continuous variables is a bad idea.**

RESPONSE
There were previous reports that the risk of thromboses after vaccination might change over time, and therefore that estimating the average HR over the whole of follow up would obscure potentially important information. We chose the time periods presented to (i) aid interpretation (since estimated ratios of hazard ratios for continuous time-dependent exposures are difficult to interpret), (ii) maintain precision (large enough number of events in each time period and (iii) examine whether HRs differed in a manner consistent with previous reports.

34. **Tables:  The formatting of the numbers isn't great. Either add commas or remove the last 3 digits and note that the numbers are in thousands. I prefer the latter, and then some numbers will have decimal parts and some won't.**

To improve formatting and retain clarity, we have added commas as suggested.

Reviewer #2: Thank you for the opportunity to review this timely manuscript on a matter of importance to public health. The size of the source data for this study is very impressive. While I commend the authors on providing a wealth of analyses on English data

35. **You state that for computational efficiency only 10% of the almost nationwide data is used. First of all, if this is correct then this is not a nationwide study. Second, how are you ensuring that the 10% selection is random; if you select individuals with equal probability, you are selecting individuals that may not have  a lot of follow-up with the same probability as individuals with complete follow-up. Third, you can only be certain that an individual is a "case" or "control" if you look at the whole follow-up period. That is, you are in some way conditioning on the future if we look at the data from a survival analysis perspective. I think you need to provide more detail on this aspect of your analysis and reassure the reader that this is a valid approach for survival data. Please also include references to where this approach has been used before. Alternatively, use another approach such as Poisson regression which should be more computational feasible for all the data.**

RESPONSE: Inverse probability (IP) weighting to account for known or estimated sampling probabilities underpins much of survey statistics and is of increasing importance in epidemiology. For example, IP weighting can be used to correct for selection bias due to informative censoring, and is the means through which marginal structural models control time-varying as well as baseline confounding. The underlying principle is that if someone had a 20% probability of being included then by giving them a weight of 5 (1/0.2) we allow them to represent the four people who were not sampled. Of course, standard errors would be too large if we pretended that their data was really that of five individuals – this problem is solved by using robust standard errors. For a detailed explanation of the approach, we suggest Chapters of Hernan and Robins’ book “What If” – see <https://cdn1.sph.harvard.edu/wp-content/uploads/sites/1268/2021/03/ciwhatif_hernanrobins_30mar21.pdf>. Section 2.4 explains the use of IP weighting to control confounding and gives a formal definition of IP weights. Chapter 12 explains the use of these weights to fit estimate causal effects based on marginal structural models.

We used IP weighting in our analyses because it was impossible to fit statistical models to the entire dataset – they simply took too long to run. We could have randomly sampled from the whole population and conducted unweighted analyses, but this would have reduced precision (there were limited numbers of some events, and in time-to-event analyses the precision of estimated effects is determined by the number of events). IP weights provide an elegant solution, which can be considered a generalization of the case-control study approach. By randomly sampling people who did not experience the outcome event and then using IP weighting in the analysis, we can analyse the data as if they came from the whole population and achieve results almost identical to those that would have been found had we been able to fit the model to the whole population. We have recently confirmed the utility of the approach by conducting simulation studies with varying sampling fractions (2%, 5% and 10%). Regardless of the sampling fraction, the results are identical on average. Because there are so many more individuals without than with the outcome event, the inflation in the standard errors is minimal.

We have clarified the text as follows: “…each fitted model used data from all individuals with the outcome of interest and a random 10% sample of all other individuals”.

36. **You should not use any stepwise selection procedure for covariate inclusion.**

RESPONSE

We prespecified and included an extensive number of known confounders. However, we wished to include as many additional potential confounders as possible. Particularly for rare outcomes, it is impossible to fit models with all known and potential confounders. Therefore for all models we included additional covariates selected in a backwards selection procedure for myocardial infarction, with a p<0.2, separately by age and sex, as specified in our published protocol. Importantly, this pragmatic approach extended the confounder adjustment in a consistent, transparent and reproducible manner, and avoided major problems in estimation caused by collinearities of confounders that would otherwise have made fitting some models impossible.

37. **Please elaborate on your approach to absolute excess risk. Is this an observed (post-vac) vs expected (historic) analysis? Please provide 95%CI also when reporting these risks.**

RESPONSE:

We have clarified the text as follows. “Age and sex-specific absolute excess risks of ICVT in days 1-28 following vaccination were calculated by multiplying estimated hazard ratios by the average monthly incidence rates of first fatal or non-fatal ICVT; the latter were derived from records during calendar year 2019 in the population-wide data resource.”

These estimates are specific to the population-wide incidence rates in England: consequently, we have not provided 95% confidence intervals (see response #1 to reviewer 1).

38. **The tables are overloaded, have formatting issues and are difficult to read.**

RESPONSE:

We have tidied up the formatting of the tables. We have presented our estimate by age and sex, which is important, because the estimates vary by these factors.

39. **Why are the main results "hidden" in a supplementary table?**

RESPONSE:

The key results are shown in figures 1-3 with full details provided in the supplementary table. Our preference is not to include the large table in the main manuscript to avoid duplication, however, we are happy to include the table in the main text if the editors wish.

40. **I am lacking a hierarchy of results. As it stands, you are presenting all possible combinations of analyses. Do all these analytical combinations represent a priori hypotheses?**

RESPONSE:

We pre-specified our analyses in our protocol. The hypothesis was

*“There is a higher risk of venous or arterial thrombosis, or haemorrhage (particularly intracranial) after COVID-19 vaccination than before or without vaccination, that varies with vaccine type, and is associated with thrombocytopenia”.*

We believe that we have addressed this hypothesis.

41. **You include fractures as a control outcome supposedly not associated with vaccination (which is not entirely clear since fractures and frailty could be associated) and present significantly protective effects of vaccination without comment? Could this protective effect not represent unadjusted confounding/bias, which would also have biased thrombotic outcome risks towards null?**
    RESPONSE:

We agree with the reviewer that the likely cause of the lower incidence of lower limb fracture after vaccination is likely to be due to residual confounding in those aged over 70. We have expanded our discussion of this as follows:

*“There were slightly lower post-vaccination rates of the ‘negative control’ outcome lower limb fracture in those aged ≥70 years, which are likely due to unmeasured confounding. This implies that* *inverse associations of vaccination with thrombotic outcomes in that age group* *may also be attributable in part to unmeasured confounding.”*

Reviewer #3: This is an interesting comprehensive analysis of the risk of venous and arterial thrombosis, or thrombocytopenia in association with ChAdOx1-S and BNT162b2 vaccination in the English adult population. I have some comments that should be addressed:

42. **The definition of the unvaccinated group is unclear. This group includes pre-vaccination times of later vaccinated persons as well as unvaccinated persons. What was the proportion of pre-vaccination times and unvaccinated persons? How was the observation time defined in the unvaccinated population? How long was the observation time? How were the controls (unvaccinated) selected? This information needs to be provided in much more detail**

RESPONSE:

In this paper, we look at the whole adult population of England from December 2020 to March 2021. Those people who were not vaccinated by the end of follow up contribute all their person-time to the unvaccinated group. For people who were vaccinated, their follow up time before vaccination is included in the unvaccinated group and their follow up after vaccination is included in the post-vaccination time periods. This is a standard approach to dealing with time-varying exposure in time-to-event analyses. There is no selection of controls, because all follow up for every individual is included in the analysis.

The reason for choosing this approach, and the problem faced by all observational studies of COVID-19 vaccination, is the rapid vaccine rollout means that there is no simple way to define a control group of unvaccinated people. For example, someone who is unvaccinated on 25 January could be considered a control, but may be vaccinated a week later. We have clarified our approach in Methods section as follows:

“*To allow for varying vaccination dates, follow up from December 8th 2020 for each person was split into periods before, and 1-28 and >28 days after, first vaccination.”*

We have added this sentence to the Results section:

“*Total unvaccinated follow up time for ages <40, 40-69 and ≥70 years was 4,529,022, 5,026,046 and 1,008,498 person-years respectively. The median follow-up per person was 101 days.”*

43. ***I*ncluding pre-vaccination times is of concern. Patients who develop VTE, ATE or thrombocytopenia would probably not get vaccinated. This introduces substantial bias.**

RESPONSE:

Please see our response to point 42 immediately above. The rapid uptake of vaccination means that it is essential to include pre-vaccination follow up in these analyses.

We agree that occurrence of venous events, arterial events or thrombocytopenia in unvaccinated people could cause them to delay vaccination, leading benefits of vaccination being overestimated. We therefore do not believe that this issue explains the association of ChAdOx1 with *higher* rates of ICVT and thrombocytopenia. Equally importantly, Table 1 shows that even in those aged >70 years the percentages who experienced venous and arterial events during the short follow up period were 0.12% and 0.66% respectively. Therefore, only a very small proportion of events would have led to postponement of scheduled vaccination, particularly in those aged <70.

44. **„…the monthly incidence of ICVT in 2019.." From where did you derive this data? Please provide information in the Methods.**

RESPONSE:

We have clarified this in the text as follows: “Age and sex-specific absolute excess risks of ICVT in days 1-28 following vaccination were calculated by multiplying estimated hazard ratios by the average monthly incidence rates of first fatal or non-fatal ICVT; the latter were derived from records during calendar year 2019 in the population-wide data resource.”

45. How did you handle missing data?

**RESPONSE:**

We defined most covariates by presence or absence of a record in the electronic health record, and therefore there was no missingness (although of course there may be a problem with accuracy of these data). There was missingness for ethnicity; here we defined a group of ‘unknown or missing’ ethnicity. Altered methods to read:

*“History of diabetes, depression, obesity, cancer, chronic obstructive pulmonary disease, chronic kidney disease, stroke, MI, DVT or PE, cancer, thrombophilia, liver disease or dementia were defined* ***as presence or absence*** *of any record in primary care and/or hospital admission data before December 8th 2020”*

46. **„Rates of intracranial venous thrombosis and thrombocytopenia in adults …" This should be „OR" rather than „AND", since you did not link the two events. In light of VITT, current presentation might be misleading particularly considering the very heterogeneous definition of thrombocytopenia. Please report thrombotic events separately from thrombocytopenia and rephrase.**

RESPONSE:
We have made this alteration and report the adjusted HR for intracranial venous thrombosis and thrombocytopenia respectively.

47. **Data on VTE and ATE risks in the general population are important to assess their risks in association with the vaccination. This would also be of interest for immunothrombocytopenia. However, the definition of thrombocytopenia in the present study is a mixed bag and includes a wide range of diseases associated with a low platelet count (e.g. TTP). It is unclear what the relevance of this data is.**

RESPONSE:

Here the data are relevant to hospitalisation or death due to thrombocytopenia (where either is reported in the primary position), and we agree that this includes a range of disease associated with low platelets. We are limited in our ability to discriminate the cases of thrombocytopenia by our available medical records.

48. **„…our primary outcome used the primary reason for death or hospital admission.." Are all DVT treated in the hospital in the UK? Is there the possibility to have missed events that were treated in an outpatient setting?**

RESPONSE:

Unfortunately, outpatient consultations were not coded in the data available to us, therefore as we have outlined in the paper, we only looked at events with hospitalisation or death. This will have under-ascertained milder events. We added the following phrase to the discussion:

*“Access to primary care data would also have improved outcome ascertainment.”*

49. **„In younger populations, who have a lower morbidity and mortality due to COVID-19, other available vaccines might be prioritise, especially when the risk of COVID-19 is otherwise low." Given the low absolute risk this conclusion cannot necessarily be drawn. The decision on the vaccine is influenced by other factors including availability and costs. Also, how would one define an otherwise low risk of COVID-19?**

RESPONSE:

We agree that the absolute benefits of risks and benefits of vaccination with ChAdOx1-S in younger people is needed; this would depend on the estimates of morbidity, mortality and chance of infection at the time of vaccination. Such a modelling exercise is outside the scope of this paper. We comment in the conclusion:

*“although more precise estimates balancing risks and benefits for adults <40 years are needed.”*
